# Emerging Lipids from Arecaceae Palm Fruits in Brazil

**DOI:** 10.3390/molecules27134188

**Published:** 2022-06-29

**Authors:** Gerson Lopes Teixeira, Elena Ibañez, Jane Mara Block

**Affiliations:** 1 Graduate Program in Food Science, Department of Food Science and Technology, Federal University of Santa Catarina (UFSC), Florianopolis 88034-001, Brazil; janeblock@gmail.com; 2 Institute of Food Science Research (CIAL), Spanish National Research Council (CSIC), Autonomous University of Madrid (UAM), 28049 Madrid, Spain; elena.ibanez@csic.es

**Keywords:** specialty oils, *Acrocomia*, *Astrocaryum*, *Attalea*, *Bactris*, *Butia*, *Euterpe*, *Mauritia*, *Oenocarpus*, *Syagrus*

## Abstract

Arecaceae palm tree fruits (APTFs) with pulp or kernel rich in oil are widely distributed in six Brazilian biomes. APTFs represent a great potential for the sustainable exploitation of products with high added value, but few literature studies have reported their properties and industrial applications. The lack of information leads to underutilization, low consumption, commercialization, and processing of these fruit species. This review presents and discusses the occurrence of 13 APTFs and the composition, physicochemical properties, bioactive compounds, and potential applications of their 25 oils and fats. The reported studies showed that the species present different lipid profiles. Multivariate analysis based on principal component analysis (PCA) and hierarchical cluster analysis (HCA) indicated a correlation between the composition of pulp and kernel oils. Myristic, caprylic, capric, and lauric acids are the main saturated fatty acids, while oleic acid is the main unsaturated. Carotenoids and phenolic compounds are the main bioactive compounds in APTFs, contributing to their high oxidative stability. The APTFs oils have a potential for use as foods and ingredients in the cosmetic, pharmaceutical, and biofuel industries. However, more studies are still necessary to better understand and exploit these species.

## 1. Introduction

The search for new sources of oils and sustainable practices in its processing, including the use of co-products, has been challenging over the last decades. Moreover, due to the high dependency on commodity oils for foods and, more recently, biodiesel production, most countries do not supply their local demands and need to import from other producers. In this scenario, Brazil stands out as one of the biggest exporters of oilseeds, including soybean, Brazil nut, and cashew. The forecasts show that the markets are progressively growing, stimulating the local producers to increase their production areas. However, these practices can contribute to higher deforestation rates, which is highly discouraged. Nevertheless, Brazil has several underutilized plant species whose fruits yield significant oil content, which may be alternatives to the few established commodities. Hence, valorizing and encouraging alternative crops for obtaining vegetable oils through government policies is urgent.

Although the global production of vegetable oils has grown since 2001 and reached 207.28 million metric tons in 2019/2020 and 206.42 million metric tons in 2020/2021, supplying vegetable oil in the current worldwide scenario has also became challenging. Furthermore, it is expected that this production will surpass 213 million metric tons in 2021/2022. The most consumed oils worldwide are based on nine crops: palm (*Elaeis guineensis*), soybean (*Glycine max*), rapeseed (*Brassica napus*), sunflower seed (*Helianthus annuus*), peanut (*Arachis hypogaea*), cottonseed (*Gossypium* L.), coconut (*Cocus nucifera*), and olive (*Olea europaea*). Among them, soybean was the leading oilseed in the 2021/2022 reference year, with 363.86 million metric tons of seeds produced globally, followed by rapeseed (70.62 million metric tons) and sunflower seed (57.26 million metric tons) [1,2]. Currently, Brazil is the leading soybean producer worldwide, with 138 million metric tons in the last marketing year (2020/2021), and it is expected to achieve 139 million metric tons in 2021/2022 [3].

**Figure 1:**
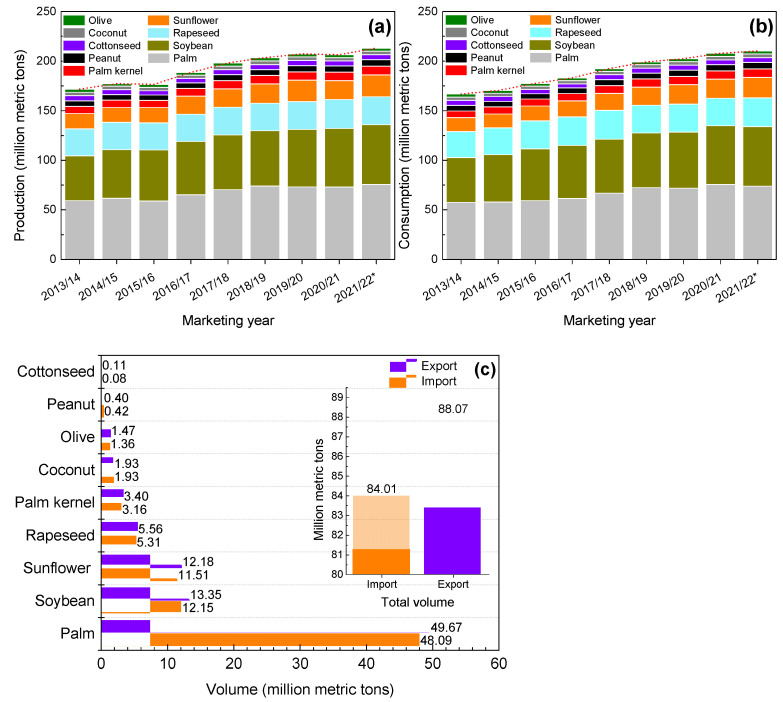
Global production (**a**) and consumption (**b**) of vegetable oils from 2013 to 2021, and the import and export volumes of major vegetable oils worldwide in the 2021/2022 marketing year (**c**). * Expected production/consumption. Adapted from Refs. [1,4,5,6]. 2022, Statista.

The worldwide production (Figure 1a) and consumption (Figure 1b) of the main vegetable oils have significantly increased between 2013 and 2021 and are expected to increase in 2022. The exportations exceeded importations in 2020/2021 (84.01 and 88.07 million metric tons, respectively), and palm oil was the leading traded vegetable oil worldwide (Figure 1c) [1,4,5,6]. These data show a growing trend in the production and consumption of commodity oils.

According to the Brazilian Association of Vegetable Oil Industries (ABIOVE), Brazil can process, refine, and bottle up more than 194,353; 23,574; and 14,612 tons of oil per day, respectively. Currently, the main oils produced in Brazil are obtained from soybean, cottonseed, sunflower, rapeseed, peanut, and castor seeds, while soybean, cottonseed, sunflower, rapeseed, coconut, palm, and corn are the foremost refined and bottled oils. The association also reports that 62 companies with 121 industrial facilities installed across the country could process vegetable oils. A total of 30 companies and 59 industrial units are ready to refine and bottle the oils. However, only 94 processing facilities and 47 refining units are currently operating, while the others are temporarily deactivated [7].

Due to the high US dollar exchange rate compared to the Brazilian currency, local oilseed producers currently prefer to export the whole grains as it is more profitable than extracting the oil. Accordingly, with the lower oil production associated with the current issues related to the COVID-19 pandemic and high local demands for vegetable oils, an increase in their prices of about five times was observed in the last two years. It reveals a vulnerability regarding the dependency on commodity oils. In addition, this scenario of ever-increasing production versus consumption may not last long, as the manufacturing capacity, planting areas, and resources for oilseeds cultivation are limited. Since the oilseeds need specific soil/climate conditions to grow, importations should increase in many countries. This high dependency on commodity oils reinforces that finding alternative vegetable oil sources is an urgent worldwide demand. Oilseeds rich in oil that can be cropped in different soils and climates have emerged in recent years, and research on their agronomic, nutritional, and processing potential is necessary.

Despite current research, Brazilian biodiversity is still little known, neglected, and underused. For instance, many palm trees found in the Amazon region, which present a high potential for exploitation, are merely used by riverside populations, indigenous people, and small farmers to produce their houses with the stems in the main structure and palm leaves to cover the roofs. Nevertheless, most of its fruits remain unexplored with scarce information on their properties, resulting in a gap in the literature to properly assess their potential for sustainable utilization.

The fruits of the palm trees from the Arecaceae family, which have high socioeconomic and ecological importance due to their wide occurrence, represent an emerging alternative as a source of oil. *Syagrus*, *Bactris*, *Attalea*, *Astrocaryum*, *Euterpe*, *Butia*, *Oenocarpus*, *Acrocomia*, and *Mauritia* are the genera of this family among the most commonly found in Brazilian biomes [8]. The seeds from those palm tree fruits have great potential for industrial applications, including in food [9,10,11,12], therapeutic and cosmetics products [11,13,14,15], and biodiesel [16,17,18,19,20,21,22]. The latter is a growing demand in Brazil due to local regulations that obligate diesel producers to include 10% biodiesel in their composition. In addition to their potential for economic exploitation, Arecaceae palms also have botanical relevance because some species are threatened with extinction [23]. A recent study on Arecaceae palms from Brazil focused on fruits from four species and their main nutritional properties, chemical attributes, and ethnobotanical characteristics [16]. However, the profile and properties of their lipid content have not been reported in the literature yet.

The occurrence and studies of thirteen Arecaceae palm tree fruits (APTFs) from the Brazilian biodiversity, the characteristics of their oils and fats, and their potential as raw materials for lipid recovery are presented and discussed in this review.

## 2. Methodology

**Figure 2:**
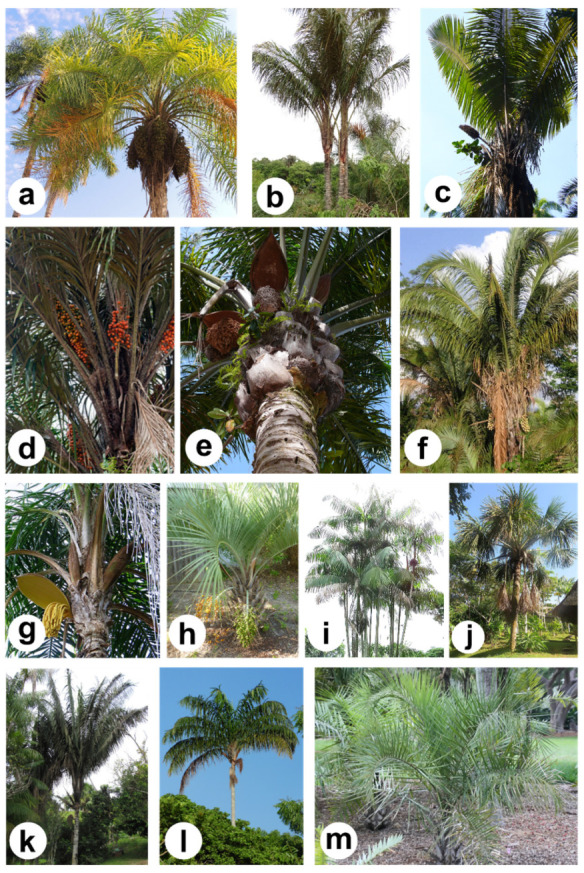
Arecaceae palm trees from Brazilian biodiversity: (**a**) *Acrocomia aculeata*, (**b**) *Astrocaryum aculeatum*, (**c**) *Astrocaryum murumuru*, (**d**) *Astrocaryum vulgare*, (**e**) *Attalea maripa*, (**f**) *Attalea speciosa*, (**g**) *Bactris gasipaes*, (**h**) *Butia* spp., (**i**) *Euterpe oleracea*, (**j**) *Mauritia flexuosa*, (**k**) *Oenocarpus bataua*, (**l**) *Oenocarpus bacaba*, and (**m**) *Syagrus coronata*. Images are royalty-free and adapted from Wikimedia Commons (http://commons.wikimedia.org/, accessed on 2 June 2022), Flickr (http://flickr.com/, accessed on 2 June 2022), Brazilian Flora 2020 [26], and iNaturalist (http://inaturalist.org/, accessed on 2 June 2022).

A total of 13 Arecaceae palm tree species (Figure 2) whose fruits (pulp or kernel) are rich in lipids were surveyed in this review. Scopus^®^ (SCO) [24], Web of Science Core Collection (WOS) [25], Brazilian Flora 2020 (BRF) [26], and Global Biodiversity Information Facility (GBIF) [8] were used as the primary databases. The scientific and common names of each species were used to find the corresponding reports and select documents related to the main subject of this study. The criteria for inclusion of the complete articles were: (1) publications in English, Portuguese, or Spanish from 1990 to 2021 and (2) studies carried out with palm trees grown in Brazilian biomes on the lipid composition; physicochemical, chemical, technological, and biological properties; and applications of their fruits. Studies focusing on the properties and applications of other parts of the plant species rather than the seeds (pulp and kernel) or cake from the lipid extraction of the target matrices, reviews, and ethnobotanical studies were excluded. In addition to the abovementioned properties, a multivariate statistical analysis based on principal component analysis (PCA) and hierarchical cluster analysis (HCA) was performed to verify possible similarities among palm tree oils and fats from the pulp and kernel, using their fatty acids (FAs) profile as input data. Brazilian states and the Federal District were coded to their corresponding acronyms: Acre (AC), Alagoas (AL), Amapá (AP), Amazonas (AM), Bahia (BA), Ceará (CE), Distrito Federal (DF), Espírito Santo (ES), Goiás (GO), Maranhão (MA), Mato Grosso (MT), Mato Grosso do Sul (MS), Minas Gerais (MG), Pará (PA), Paraíba (PB), Paraná (PR), Pernambuco (PE), Piauí (PI), Rio de Janeiro (RJ), Rio Grande do Norte (RN), Rio Grande do Sul (RS), Rondônia (RO), Roraima (RR), Santa Catarina (SC), São Paulo (SP), Sergipe (SE), and Tocantins (TO).

## 3. Occurrence, Distribution, and Studies on Arecaceae

**Figure 3:**
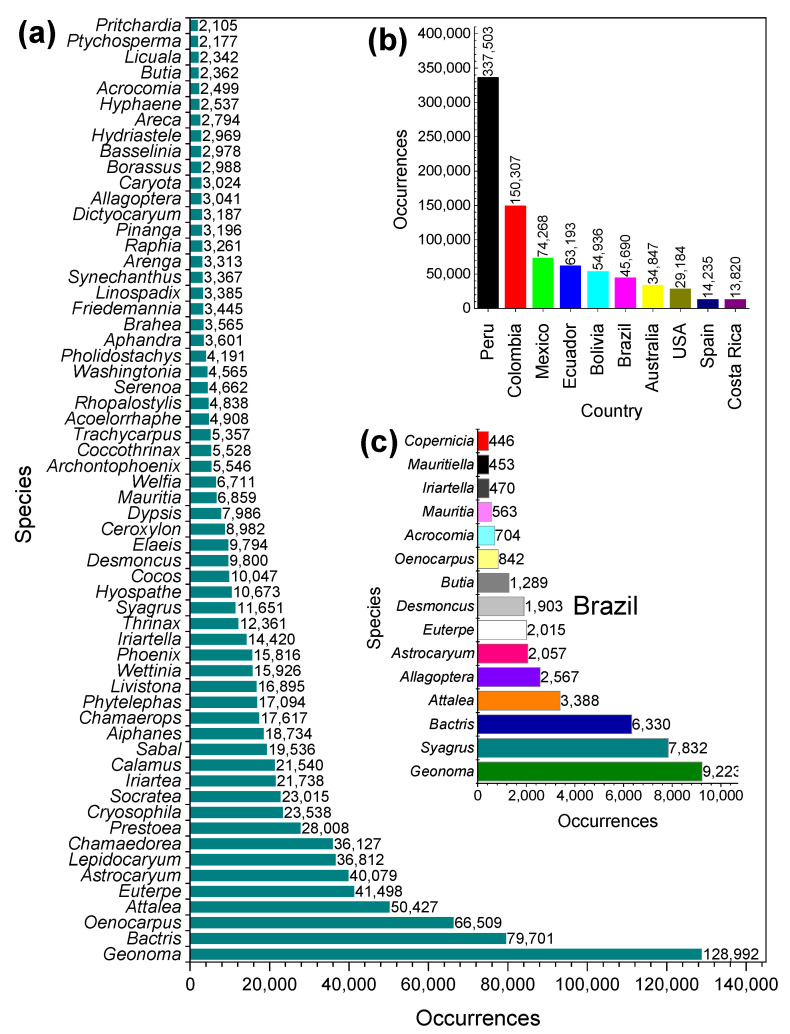
Occurrence of Arecaceae species around the globe. The 60 species with higher occurrence worldwide (**a**); the 10 countries with the highest rate of Arecaceae species (**b**), and the 15 main Arecaceae species found in Brazil (**c**). Adapted from Ref. [8].

The GBIF database [8] is a platform that provides a broad range of data related to the reported occurrences of many plant species. Currently, the platform shows that the Arecaceae family has 3819 species cataloged worldwide. Figure 3a depicts the top 60 species of highest occurrence. The main prominent species, among the 1,002,528 occurrences of species associated with this family reported in the database, are *Geonoma* (13%), *Bactris* (8%), *Oenocarpus* (7%), *Attalea* (5%), and *Euterpe* (4%). More than 93% of palm plant species can be found in the American continent, mainly in South America (83%) and North America (10%). The highest occurrence rate of Arecaceae palms is observed in Peru (41%), followed by Colombia (18%), Mexico (9%), Ecuador (8%), Bolivia (7%), and Brazil (6%) (Figure 3b). On the other hand, Australia, the USA, Spain, and Costa Rica account for 12% of the total occurrences of Arecaceae palms. In Brazilian biomes, 37 genera and about 300 Arecaceae species occur naturally [27], with a high incidence of *Geonoma* (23%), *Syagrus* (20%), *Bactris* (16%), *Attalea* (8%), *Astrocaryum* (5%), *Euterpe* (5%), *Butia* (3%), *Oenocarpus* (2%), *Acrocomia* (2%), and *Mauritia* (1%) (Figure 3c) [8].

**Figure 4:**
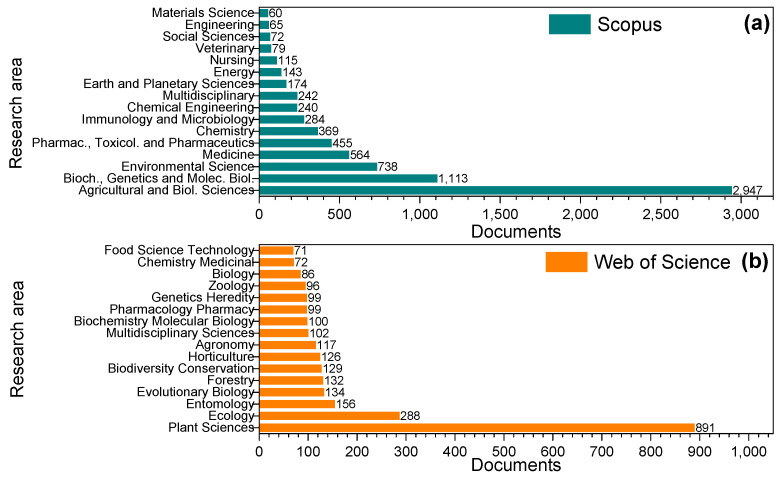
Studies related to Arecaceae on Scopus^®^ (**a**) and Web of Science (**b**) databases ranked by their main research areas from 1990 to 2021. Adapted from Refs. [24,25]. Accessed on 2 June 2022.

Figure 4 shows the number of documents and the main research areas associated with “Arecaceae” on Scopus (SCO) and Web of Science (WOS) platforms between 1990 and 2021. The data demonstrate that the number of records in the mentioned period in SCO (4808, accessed on 2 June 2022) is twice that of the WOS (2271, accessed on 2 June 2022) database. The study areas are differently classified in each platform as well. This massive difference can be attributed to the mechanisms and algorithms from the engine search of each database. Such discrepancies reinforce the use of different archives for a proper survey of each raw material. The records in SCO show that the documents related to Arecaceae are research articles (90.8%), reviews (4.2%), and conference papers, book chapters, short surveys, books, and others (5%). Conversely, WOS indicates 87.5% articles, 5.9% proceeding papers, 3.7% reviews, and 2.9% editorials, letters, notes, and datasets. Figure 4a demonstrates that the publications with Arecaceae palms retrieved from SCO are mostly linked to Agricultural and Biological Sciences (37.3%), followed by Biochemistry, Genetics, and Molecular Biology (14.1%), Environmental Sciences (9.3%), Medicine (7.1%), Pharmacology, Toxicology, and Pharmaceutics (5.8%), and Chemistry (4.7%). On the other hand, documents in WOS (Figure 4b) are mainly related to Plant Sciences (39.2%), Ecology (12.8%), Entomology (6.9%), Evolutionary Biology (5.8%), and Forestry (5.6%). Food Science and Technology accounts for 3.1% of related documents. Most studies on Arecaceae in SCO come from Brazil, followed by the USA and Malaysia (24.7, 16.0, and 10.1%, respectively). Similarly, WOS shows Brazil (32.1%) and the USA (20.2%) as the leading countries that research Arecaceae species, followed by England (7.7%), France (7.3%), Denmark (6.9%), and India (5.6%).

Information regarding non-conventional raw materials and specific plant species retrieved from databases must be evaluated carefully as some divergencies usually appear. In addition, the different database patterns to classify the documents typically return a different number of studies, and some reports could be missed when using a single platform to retrieve the data. Thus, it is recommended to use as many platforms and databases as possible for such studies.

**Figure 5:**
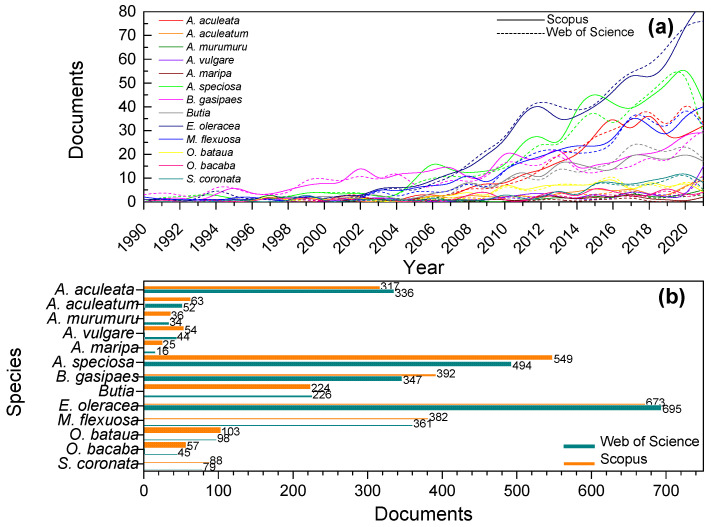
The evolution of studies related to 13 different Arecaceae palm trees on Scopus^®^ and Web of Science (**a**) and a comparison of total documents in each database between 1990 and 2021 (**b**). Adapted from Refs. [24,25]. Accessed on 2 June 2022.

Figure 5a shows the evolution of documents in the SCO and WOS databases related to the APTFs. A growing trend in the number of publications is observed for some of them. On the other hand, some APTFs present few studies in the twenty-year range surveyed. A difference in the total scientific papers obtained through the SCO and WOS platforms from 1990 to 2021 can be noted (Figure 5b). However, in both databases, the highest number of documents were associated with *E. oleracea* (673–695), followed by *A. speciosa* (494–549), *B. gasipaes* (347–392), *M. flexuosa* (361–382), and *A. aculeata* (317–336), while the species with the lowest number of documents in the literature were *A. maripa* (16–25) and *A. murumuru* (34–36). Also, the highest discrepancies between the results from each database were observed in *B. gasipaes* (13%) and *A. speciosa* (10%).

**Figure 6:**
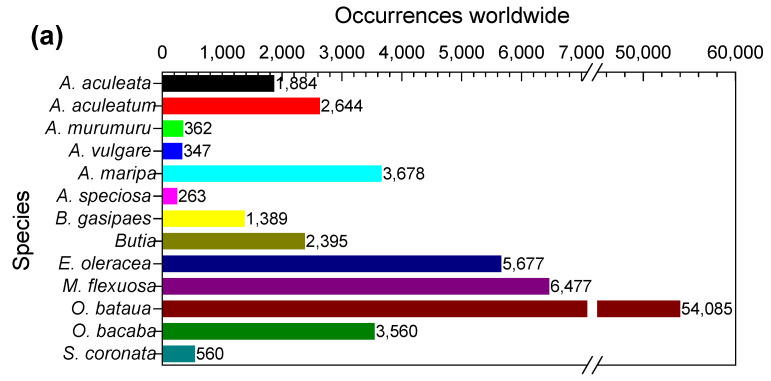

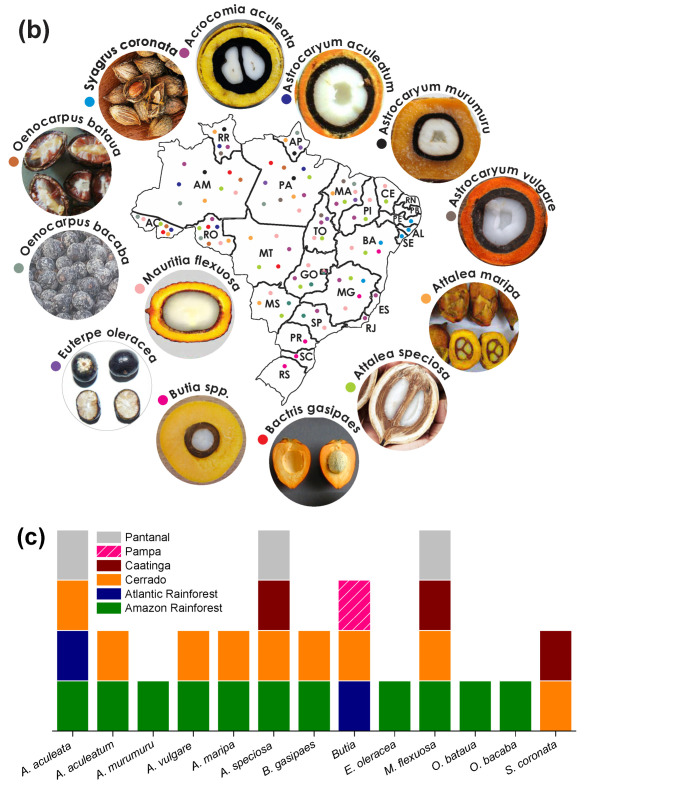
Occurrence of 13 Arecaceae palm trees worldwide (**a**) and their fruits with the corresponding presence in Brazilian states (**b**) and Brazilian biomes (**c**). Adapted from Refs. [8,26]. Fruit images are royalty-free, adapted from Brazilian Agricultural Research Corporation—EMBRAPA (http://embrapa.br/imagens/, accessed on 2 June 2022) and Wikimedia Commons (http://commons.wikimedia.org/, accessed on 2 June 2022).

Figure 6a shows the global occurrences of 13 Arecaceae palm trees, and Figure 6b,c show the incidence of APTFs in Brazilian states and biomes, respectively. The Brazilian states PB and RN have no records of such palm trees. On the other hand, at least one of them is found in the other 24 states and the Federal District [27]. The data indicate that *A. aculeata*, *A. speciosa*, and *M. flexuosa* are the palm trees with the highest occurrence rate in Brazilian biomes. According to the available literature, species such as *Acrocomia aculeata*, *Attalea speciosa*, *Euterpe oleracea*, *Mauritia flexuosa*, and *Syagrus coronata* have been largely cultivated in Brazil, and their products were the most studied among the 13 species reviewed in this study. Thus, they could be the most promising palm plant species for cultivation for commercialization.

## 4. Arecaceae Palms in Brazil

Table 1 shows 13 emerging Arecaceae palm trees, their scientific and common names, the part of the plant where the oil can be extracted, oil content, and the code used in this review. Since most APTFs provide oil from the pulp and kernel, different coproducts are generated from a single fruit.

The APTFs grown in Brazil are emerging crops that can produce alternative oils and fats. Despite their high occurrence and significant oil content, their processing can be challenging. The use of obsolete machinery in small factories provides low yields and generates tons of waste that are not adequately used, causing valuable losses. Some of these oils are sold in specific regional markets or online stores and can be used in dressings, salad oils, margarine, nanoemulsions, biofuels, shampoos, soaps, moisturizing agents, repellents, conditioners, and skin cleaners. Despite being used in many commercial products, these oils are still little known and studied.

### 4.1. Acrocomia aculeata

*Acrocomia aculeata* ((Jacq.) Lodd. ex R. Keith) is mainly found in Mexico and Brazil. It has also been found in 33 different countries, islands, and other territories worldwide, such as Colombia, Costa Rica, Bolivia, Argentina, the United States of America, Jamaica, Paraguay, Venezuela, Puerto Rico, Guatemala, Nicaragua, the Dominican Republic, Trinidad and Tobago, Honduras, Panama, Cuba, Martinique, Suriname, Belize, French Guiana, El Salvador, Haiti, Dominica, Guadeloupe, Guyana, Indonesia, Saint Vincent and the Grenadines, Germany, Grenada, Saint Kitts and Nevis, Sri Lanka, Thailand, and Uruguay [8]. The palm tree of *A. aculeata* reaches 10 to 15 m in height [18]. The genus presents a wide distribution due to its high adaptability to many soil and climate conditions. There are 1884 occurrences of *A. aculeata* reported in Northern, Central, and South America (Figure 6a) [8].

Most studies involving *A. aculeata* in WOS are related to Agronomy (16.9%), Plant Sciences (16.1%), Agricultural Engineering (10.7%), and Food Science and Technology (11.0%) [25]. On the other hand, SCO indicates Agricultural and Biological Sciences (39.5%), followed by Biochemistry, Genetics, and Molecular Biology (10.7%), and Environmental Science (7.7%) [24]. The domestication of this species in Brazil started in 1983 by the Brazilian Agricultural Research Corporation (EMBRAPA). The studies indicated a high potential as the palm tree was easily found in pastures, disturbed areas, tilled land, and road margins [70].

The fruits of *A. aculeata*, known as macaúba, mucajá, and coco-babão, have been registered in 14 states (AM, BA, ES, FD, GO, MA, MG, MT, PA, PI, RJ, RO, RR, TO, and SP) covering four biomes: the Amazon, Atlantic rainforest, Cerrado, and Pantanal (Figure 6b,c) [71]. Since the oil can be extracted from both the pulp and kernel [17], macaúba is considered an emerging alternative crop to palm (*E. guineensis*) oil. It can become a commercial oilseed crop [22,72] and alternative raw material for oil extraction [17,73], bioenergy [74], and biofuel production [73,75,76]. After many efforts and massive research over the last four decades, *A. aculeata* was adapted for cultivation in some regions of Brazil. Its fruits are currently used industrially to obtain vegetable oils and charcoal from residues. The species has been reported as a neotropical “green gold” as it provides many plant products, such as oil, wax, proteins, carbohydrates, fibers, and other coproducts [17]. Macaúba is a multipurpose palm tree [72] with high productivity, reaching up to 62 kg per plant [70] and about 25 tons per hectare [20].

In 2018, the Ministry of the Environment of Brazil started the so-called “Macaúba Project”. This is a pilot project that is a part of the Forest Investment Program funded by the Inter-American Development Bank. The project’s main goal, which has a budget of USD 3,000,000.00, is to develop an alternative to palm oil by consolidating the productive chain of macaúba as a source of sustainable vegetable oils. The project includes research on seedlings, planting techniques, and handling in the silvopastoral system, and developing industrial machinery for processing macaúba. The target area is the Cerrado (Alto Paranaíba, MG, Brazil), where family farmers have cultivated 2000 hectares of macaúba. In addition, the project is promoting the extractive collection of up to 1500 tons of native palm fruits per year [77].

Macaúba palm tree fruits are yellow-greenish, and the pulp is used in culinary preparations [18]. In addition, those fruits are rich in lipids, vitamins, and minerals [78,79].

### 4.2. Astrocaryum aculeatum

The genus *Astrocaryum* has 40 species distributed in South America, and more than 40,308 occurrences registered. In Brazil, 26 species of *Astrocaryum* are found. The *Astrocaryum aculeatum* (G. Mey.) species has 2644 occurrences reported worldwide (Figure 6a), most of them in Colombia (1786) and Brazil (462) [8]. This palm tree occurs in Northern (AC, AM, PA, RR) to Midwestern (MT) states (Figure 6b), extending its distribution from the Amazon rainforest to Cerrado biomes (Figure 6c) [80]. Most research involving *A. aculeatum* registered in WOS is related to Food Science and Technology (25.5%), Plant Sciences (13.2%), Agronomy (9.43%), and Ecology (9.43%) [25]. Moreover, Agricultural and Biological Sciences (41.2%) are the primary research areas in SCO, followed by Biochemistry, Genetics, and Molecular Biology (12.7%) and Chemistry (9.8%) [24].

The *Astrocaryum aculeatum* is popularly named tucumã-do-Amazonas or simply tucumã and is often confused with *Astrocaryum vulgare* due to their similarities. *A. vulgare* is another native palm tree addressed in this study, also called tucumã. The tucumã-do-Amazonas palm tree reaches about 25 m in height and 40 cm in diameter, with internodes covered by black spines up to 25 cm long [80]. This palm tree is usually found in Manaus (AM), growing in many pastures and fields. The average yearly production ranges between two and seven bunches of 156–240 fruits showing wide morphological characteristics variability [81]. They are globe-shaped to ellipsoid with a smooth yellowish epicarp with a length of 41.5–61.7 mm, diameter of 34.3–51.1 mm, and an average weight of 47.5 g. The fruit presents a hard and black endocarp and an orange mesocarp (pulp) with a thin layer (2.5–5.2 mm thickness) as the main edible part of the fruit. It also presents a homogeneous and white endosperm [80,81]. The fruits are eaten fresh as a part of breakfast [34,35] and the pulp is used as an ingredient to prepare juice and ice cream [81], mainly in Manaus (AM), where they are sold downtown in the local markets and street fairs [34,35].

The consumption of tucumã-do-Amazonas has been associated with an increase in longevity and the reduced prevalence of age-related morbidity in the population of riverside communities in the Amazon region [82]. The seed represents about 70% of the fruit weight and is usually discarded after pulp removal, generating about 272 tons per year of waste in Manaus. Currently, the waste has no economic value, causing financial and environmental losses [35].

### 4.3. Astrocaryum murumuru

The palm tree of murumuru may reach 15 m in height and 15–30 cm in diameter [83]. The current records on GBIF [8] show 362 occurrences of *A. murumuru* in South America (Bolivia, Brazil, Colombia, Ecuador, Peru, and Venezuela) (Figure 6a). The distribution of this palm tree in Brazil is limited to four states (AP, AM, PA, and RR; Figure 6b) in the Amazon biome (Figure 6c) [83]. The studies related to *A. murumuru* in the WOS database are mainly associated with Ecology (32.4%), Plant Sciences (11.8%), Agriculture, Dairy, and Animal Science (8.8%), Agriculture Multidisciplinary (8.8%), and Biodiversity Conservation (8.8%) [25]. In addition, SCO shows Agricultural and Biological Sciences (51.0%) as the primary subject grouping, followed by Biochemistry, Genetics, and Molecular Biology (7.8%), Environmental Sciences (7.8%), and Pharmacology, Toxicology, and Pharmaceutics (8.9%) [24].

The fruits of murumuru are an inverted cone laterally flattened, 6–8.5 cm long × 3.8–4.4 cm in diameter [83]. They present a creamy pale orange pulp that covers the wood-encased nut. The pulp is sweet and tasty, and the orange color suggests the presence of carotenes [38]. Also described as yellowish, murumuru pulp has little commercial value, serving only as food for animals such as bovines [84]. The murumuru tree provides edible kernels, pulpy mesocarp, heart-of-palm, livestock feed, and grubs that feed on decaying trunks [38]. For every 100 kg of dry seeds, about 27–29 kg of *A. murumuru* kernels can be obtained [84]. The chemical profile of the murumuru pulp and its oil are not extensively known yet. This fact may be related to its great perishability since it rots quickly after the fruit falls to the ground [36].

Recently, agro-industries and entrepreneurs have established partnerships to extract the fat from 220 tons of murumuru harvested in Afuá (PA) in northeastern Brazil [85]. The thermal properties [10], the content of bioactive compounds [11], the antibacterial activity [85], and the interesterification [86] of the murumuru butter have been studied.

### 4.4. Astrocaryum vulgare

The *Astrocaryum vulgare* (Mart.) is a palm tree similar to *A. aculeatum*. The database from GBIF [8] indicates 347 occurrences of *A. vulgare* species in South America (Figure 6a). Its distribution in Brazil comprises five states (AM, GO, MA, PA, and TO; Figure 6b) from the Amazon rainforest to Cerrado biomes (Figure 6c) [87]. Most studies in WOS on *A. vulgare* are related to Food Science and Technology (31.8%), Applied Chemistry (18.2%), Chemical Engineering (9.1%), and Plant Sciences (9.1%) [25]. Conversely, SCO shows that the studies are related to Agricultural and Biological Sciences (36.4%), followed by Chemistry (20.5%), Biochemistry, Genetics, and Molecular Biology (10.2%), and Pharmacology, Toxicology, and Pharmaceutics (10.2%) [24].

*A. vulgare* is known as tucumã-do-Pará, tucum-piranga, tucum-bravo, or tucumã [87]. Vernacular names also include aoura (French), awara palm, tucum palm, tucuma palm (English), and cumare (Spanish) [8]. This palm tree, which has an average height and diameter of 20 m × 20 cm, is native to Northern and Midwestern Brazil [87]. The fruits are 3.5–5 cm in length × 2.4–4 cm in diameter and weigh 22–45 g. The mesocarp, epicarp, and endocarp have an average weight of 5.68, 6.75, and 10.03 g, representing 25.0, 29.4, and 45.6% of the fruit, respectively [40,88]. The fruits of *A. vulgare* present about 53% pulp yield, while its kernel represents 20–24% of the fruit [37,39].

### 4.5. Attalea maripa

The *Attalea maripa* ((Aubl.) Mart.), called inajá or maripa palm, has ambiguous scientific nomenclature, making searching for data related to this species complex. The literature indicates both *Maximiliana maripa* ((Aubl.) Drude) and *Attalea maripa* ((Aubl.) Mart.) as the scientific name of this plant species. Between the 3678 occurrences in the GBIF database (Figure 6a), 96.2% of the records with inajá indicate its scientific name as “*Attalea maripa*”. A few results (2.8%) are currently displayed in the database when searching for “*Maximiliana maripa*” [8]. Although there are 22 synonyms attributed to this palm tree, the Brazilian Flora 2020 project confirmed “*Attalea maripa*” as the correct name of inajá [89]. The differences in the nomenclature can make it difficult to research in the databases.

WOS displays more scientific documents using “*Maximiliana maripa*” as the entry. Most research studies involving the inajá palm tree in the WOS platform are related to Food Science and Technology (52.6%), Applied Chemistry (31.6%), Ecology (15.8%), Physical Chemical (10.5%), and Chemical Engineering (10.5%) [25]. On the other hand, SCO retrieves more documents providing “*Attalea maripa*” as the entry. In this platform, Agricultural and Biological Sciences (58.8%) are the primary research areas related to *A. maripa*, followed by Environmental Sciences (14.7%), Biochemistry, Genetics, and Molecular Biology (5.9%), and Chemistry (5.9%) [24].

*A. maripa* is widely distributed in South America in Brazil, Bolivia, Colombia, Ecuador, Guyana, French Guyana, Suriname, Peru, and Venezuela. Figure 6b,c show that *A. maripa* occurs in six Brazilian states of the Amazon biome (AC, AM, AP, PA, RO, and RR) and three of the Cerrado (MA, MS, MT). Vernacular names for this species comprise königspalme (German), inajá, maripa, najá (Portuguese), and maripa palm (English) [8]. *A. maripa* is a tall palm tree with a 5–25 m height and a solitary stem of 1–20 × 0.2–0.50 m. The species is easily recognized by its distributed leaves on the stem in a helical manner in five clear vertical lines [89]. Inajá palm tree has socioeconomic relevance due to its many valuable products (fruits, stems, palm heart), mainly the fruits and kernel, which are eaten fresh or as snacks [90].

The fruits from *A. maripa* palm are ellipsoids measuring 3.9–6.0 × 1.8–3.5 cm. The fruits have a brown epicarp, ellipsoid, and bony endocarp with smooth surfaces, without longitudinal fiber marks. The inajá is one of the few *Attalea* spp. whose mesocarp can easily be separated from the endocarp [89]. Inajá fruits provide edible pulp and kernel, which can be consumed uncooked or used to obtain edible oil [43]. Inajá palm tree has significant production levels (up to 108 kg fruits/plant), indicating its suitability as an oil crop [42].

### 4.6. Attalea speciosa

Babassu, or babaçu, is the common name for *Attalea speciosa* (Mart. ex. Spreng), a palm tree from Brazil and Colombia [8]. It is also known for its synonyms *Orbignya phalerata* (Mart.), *Orbignya speciosa*, and *Orbignya oleifera*. Due to these ambiguous names, Cavallari and Toledo [91] performed a systematic study on the ascribed names and concluded that *Attalea speciosa* suits better to babassu tree than the others, a choice further supported by the BRF project [92].

The terms “*Attalea speciosa*”, “*Orbignya phalerata*”, “*Orbignya oleifera*”, “*Orbignya* spp.”, “babassu”, and “babaçu” were used for the research in the scientific literature in the SCO platform. The term “babassu” presented the highest records, with 546 results, while *Orbignya*, babaçu, *Orbignya phalerata*, *Attalea speciosa*, *Orbignya speciosa*, and *Orbignya oleifera* returned 278, 135, 83, 76, 52, and 11 results, respectively [24]. Duplicates have been removed from the number of available documents to evaluate the studies.

The research in the GBIF platform using *Attalea speciosa* as the entry resulted in 263 occurrences of this palm species in South America (Figure 6a) [8]. There is a wide distribution of *A. speciosa* in 13 states in Brazil (AC, AM, BA, CE, GO, MA, MG, MS, MT, PA, PI, RO, and TO) along four biomes: the Amazon, Cerrado, Caatinga, and Pantanal (Figure 6b,c) [92]. Most research involving *A. speciosa* (term used: babassu) in the WOS is related to Chemical Engineering (15.1%), Biotechnology and Applied Microbiology (9.7%), and Energy and Fuels (9.0%) [25]. SCO indicates Agricultural and Biological Sciences (18.9%) as the main tag subject, followed by Chemistry (15.2%) and Chemical Engineering (11.9%) [24].

Babassu, one of the most important palms in Brazil [93], has great socioeconomic and ecologic importance and is mainly found in humid tropical areas, especially in frequently burned and degraded landscapes [94]. The parts used include the straw (used to cover houses as a rooftop), fruits, and seeds. This palm tree can reach up to 30 m in height and presents a large stalk. It is one of the most economically important species in northeastern Brazil, especially in the Maranhão state [95]. The fruits, also known in Brazil as babaçu coconut, are classified as a drupe that hangs from bunches presenting four per tree per season, with 15–25 coconut fruits each; they are oblong-shaped, measuring about 6 cm, comprising between two and six seeds [14,95].

### 4.7. Bactris gasipaes

*Bactris gasipaes* (Kunth) is a palm tree up to 20 m in height common in South America. The ripe fruits are reddish, orange, or yellowish, ovoid to subglobose, with a size of 3–6 × 2.5–4.0 cm. The pulp is abundant, starchy, floury, and oily and is very appreciated by Amazonian populations [96]. The palm tree is cultivated in some regions of Brazil to extract the palm hearth [97]. Vernacular nomenclature consists of pupunha (Portuguese), persikopalm (Swedish), and peach palm (English) [8].

Data from GBIF [8] indicate 1389 occurrences of the *B. gasipaes* palm tree species in Central and South America (Figure 6a). The distribution of peach palm across Brazil includes five states (AC, AM, PA, RO, and MT; Figure 6b) and the Amazon and Cerrado biomes (Figure 6c) [96]. The studies involving *B. gasipaes* in the WOS are associated with Plant Sciences (19.8%), Agronomy (15.2%), and Food Science and Technology (14.9%) [25]. On the other hand, SCO indicates Agricultural and Biological Sciences (51.1%) as the main linking tag, followed by Biochemistry, Genetics, and Molecular Biology (8.2%) and Environmental Sciences (8.0%) [24].

### 4.8. Butia spp.

The genus *Butia* (Becc.) Becc. is a group of palm trees widely found in South America [98]. It is an endemic native species to Southern Brazil and Uruguay [99]. GBIF [8] shows 24 species cataloged and associated with *Butia* palm trees. The database also reports more than 2395 entries of *Butia* spp. across the globe (Figure 6a). In Brazil, 19 species of *Butia* can be found in eight Brazilian states (BA, GO, MG, MS, SP, PR, RS, SC) and the Federal District, covering three biomes (Figure 6b,c) [100]. Although presenting a wide distribution in Brazil, studies on the sustainable management, processing, added value, and preservation of butiá fruit and its pulp are still scarce [101].

The studies involving *Butia* spp. In WOS are majorly related to Plant Sciences (26.3%), Food Science and Technology (16.8%), and Horticulture (9.5%) [25]. SCO indicates studies on *Butia* related to Agricultural and Biological Sciences (49.4%), Biochemistry, Genetics, and Molecular Biology (9.8%), and Environmental Sciences (8.6%) [24].

Widely distributed in Southern Brazil, the *Butia* genus is an essential part of Rio Grande do Sul biodiversity, where at least five species occur [23,102]. Among them, *Butia capitata* has shown industrial and technological potential. Its fruits are consumed fresh and processed [103], such as juices and liqueurs. The development of products from its fruits represents a sustainable alternative use for this important palm tree and income for the population [102]. The species of *B. exilata* (Deble & Marchiori), *B. lallemantii* (Deble & Marchiori), *B. catarinensis* (Noblick & Lorenzi), *B. eriospatha* ((Mart. ex Drude) Becc.), *B. odorata* ((Barb. Rodr.) Noblick), *B. paraguayensis* ((Barb. Rodr.) L. H. Bailey), *B. witeckii* (K. Soares & S. Longhi), and *B. yatay* ((Mart.) Becc.) can also be found in Rio Grande do Sul state [104,105]. Native to the Atlantic rainforest, *B. eriospatha* has suffered severe population decline due to the reforestation of exotic tree species, illegal sale of adult plants in local and international trade, overexploitation of its fruits, and herbivory due to local livestock farming [106].

*B. capitata* is known as “coquinho-azedo” and plays an important socio-economic role in poor communities in northern Minas Gerais in the Cerrado biome. The fruits are very appreciated for consumption in natura. The frozen pulp is used to prepare juices, jellies, and ice-creams. *B. capitata* fruits are employed as animal feed as well [107,108]. The seed, a residue from the processing of frozen pulp, is rich in fat [52]. However, with the expansion of agricultural frontiers and intense extraction, this species has been reported as threatened with extinction [109].

*B. odorata* palm tree might produce up to six bunches per year, and its fruits are used as ingredients for pastries, ice cream, and drinks [98]. *B. catarinensis*, which is endemic to the southern coast of Brazil, is a small palm with a 2 m height. The production rate can be as high as 927 kg of fruit and 190 kg of pulp ha^−1^. Local farmers have the extractive activity as a complement to their income, using the fruit and pulp to produce juice, wine, rum, popsicles, ice cream, and candies [110]. The *B. purpurascens* (Glassman) species occurs mainly in southwestern Goiás. This species has also been enlisted as a critically endangered species in Brazilian Flora’s Official National List of Endangered Species [111].

Due to its potential for sustainable exploitation, many scientists have put their efforts into researching alternative uses for *Butia* fruits. However, some concerns were raised due to the decreasing number of those palm trees in Southern Brazil, where it is slowly disappearing from the landscape due to human activities and urban and agricultural expansion [98,106].

### 4.9. Euterpe oleracea

The Amazonian native *Euterpe oleracea* (Mart.) palm tree reaches 3–20 m in height and 7–18 cm in diameter and presents an edible palm heart [112]. Vernacular names include açaí, açaí-do-Pará, açaizeiro, palmito-açaí, uaçaí (Portuguese), kohlpalme (German), asaí, euterpe (Spanish), assai palm, and cabbage palm (English) [8]. Açaí fruits are rounded or ellipsoid-shaped, 1–2 cm in diameter, with a smooth epicarp whose color changes from green while unripe to a blackish-purple when ripe. The thin mesocarp yields a viscous purple-wine pulp [112]. *E. oleracea* palm tree is one of the most productive of its group, and the pulp is widely used to produce wine, juices, ice cream, jelly, and confectionery [113].

There are 5677 occurrences of *E. oleracea* in South America (Figure 6a) tagged on GBIF [8]. In Brazil, the species occurs spontaneously in four states (AP, MA, PA, and TO; Figure 6b) in the Amazon and Cerrado biomes (Figure 6c), establishing large clusters close to rivers [112]. Açaí is currently known globally due to the popularization of its pulp, which can be stored frozen for long periods, facilitating transportation and spreading across the globe. The scientific literature related to *E. oleracea* in the WOS platform is mainly associated with Food Science and Technology (29.4%), Nutrition and Dietetics (13.3%), and Plant Sciences (9.7%) [25]. The database SCO indicates Agricultural and Biological Sciences (31.2%) as the primary research category, followed by Medicine (11.2%) and Biochemistry, Genetics, and Molecular Biology (10.4%) [24].

### 4.10. Mauritia flexuosa

*Mauritia flexuosa* (L. f.) is a palm tree that is 3–25 m in height × 23–80 cm in diameter, which occurs naturally in Brazil [114]. This plant species shows the following vernacular names: buriti, buritirana, caraná, caraná-do-mato, miritirana (Portuguese), morichepalme, yurumabrot (German), itapalm (Swedish), and tree-of-life (English) [8]. The tree produces from 1 to 10 bunches, supporting branches with 450–2000 fruits per cluster [57,115].

The fruits are ellipsoid-oblong, with a 3.5–5.5 cm diameter, and have an epicarp covered by overlapping scales of about 6 mm (reddish-brown in color), yellow-orange fleshy mesocarp, homogenous endocarp, and endosperm [114]. The small covering scales come off easily when the fruit is ripe, in the presence of water [57]. *M. flexuosa* fruit weight varies between 15 and 75 g. On average, bunches with 800 fruits of 50 g may yield 40 kg of fruits, achieving 160 kg of fruit per tree in a good harvest [115]. The stipe is used in construction and furniture, while the internal part is processed into flour to produce bread and porridges. The leaves are utilized to cover houses, the petiole is a raw material for making toys, and the fruits are eaten fresh or processed into, e.g., juices, wines, jams, cakes, ice cream, sweets, and flour. It has ornamental potential as well [114].

GBIF [8] indicates 6477 occurrences of *M. flexuosa* in South America (Figure 6a) [114]. The occurrence of buriti palm in Brazil has been recorded in 14 states (AC, AM, BA, CE, GO, MA, MG, MS, MT, PA, PI, RO, SP, and TO) and the DF (Figure 6b), covering the Amazon, Cerrado, Caatinga, and Pantanal biomes (Figure 6c) [114]. The documents citing *M. flexuosa* in WOS are related to Food Science and Technology (17.2%), Plant Sciences (11.9%), and Ecology (11.1%) [25]. In comparison, SCO shows Agricultural and Biological Sciences (35.81%), followed by Environmental Sciences (12.6%) and Biochemistry, Genetics, and Molecular Biology (7.8%) [24].

Most studies on *M. flexuosa* fruit are related to its pulp, which has an orange color due to its high carotenoid content, mainly β-carotene [116,117]. A report on evaluating the potential of the buriti kernel for the production of activated carbon was also found [118].

### 4.11. Oenocarpus bataua

*Oenocarpus bataua* (Mart.) is one of the most common palm trees in South America. This palm tree has a solitary, erect, and usually columnar smooth stem, 5–25 m high and 20–45 cm in diameter. Its fruits are oblong, 2.7–4.5 cm long, and dark purple with a ruminated endosperm [119]. The palm tree is named patauá or patoá (Portuguese); sejepalme (German); seje and ungurahui (Spanish); and bataua palm and kumbu (English) [8].

The database GBIF [8] indicates that *O. bataua* has a wide distribution and the highest number of occurrences (54,085) among the Arecaceae palm trees described in this study (Figure 6a). This species is distributed in four Brazilian states (AC, AM, PA, and RO; Figure 6b) in the Amazon biome (Figure 6c) [119]. Studies involving *O. bataua* in the WOS database are mostly related to Ecology (22.4%), Plant Sciences (19.4%), and Food Science and Technology (14.3%) [25]. SCO indicates that almost half of the studies are linked to Agricultural and Biological Sciences (49.0%), followed by Biochemistry, Genetics, and Molecular Biology (10.3%) and Environmental Sciences (7.7%) [24].

Most studies involving *O. bataua* are dedicated to assessing its pulp. It has been reported that the seeds of patawa are rich in phenolic compounds [120] and the oil obtained from its pulp is stable to the oxidation [11].

### 4.12. Oenocarpus bacaba

*Oenocarpus bacaba* (Mart.) is a palm tree with a 7–20 m height and 15–25 cm diameter. Its fruits are ellipsoid, 1.3–1.5 cm long, and dark-purple colored with a single seed. *Oenocarpus bacaba* var. grandis (Burret) Wess. Boer, *Oenocarpus grandis* Burret, *Oenocarpus hoppii* Burret, and *Jessenia bacaba* (Mart.) Burret are its reported synonyms [121]. Common nomenclatures include bacaba, bacaba-açu (Portuguese), and bacaba palm (English) [8]. There are 3560 occurrences of *O. bacaba* in South America (Figure 6a), as reported by the GBIF [8]. Although scientific reports indicate the occurrence of bacaba in five Brazilian states (AC, AM, AP, PA, and RO; Figure 6b), the species can also be found in the Maranhão state. The distribution of *O. bacaba* is mainly in the Amazon and Cerrado biomes (Figure 6c) [121].

The reports on *O. bacaba* in the WOS are related to Food Science and Technology (28.9%), Ecology (20.0%), Plant Sciences (20%), and Applied Chemistry (15.6%) [25]. On the other hand, SCO indicates Agricultural and Biological Sciences (48.0%), Environmental Sciences (14.0%), and Chemistry (10.0%) as the top subjects [24]. Like açaí, the fruits obtained from the *O. bacaba* palm tree present economic potential due to their processing as pulp and wine. The pulp shows a yield per fruit ranging from 34 to 38% [37,122].

### 4.13. Syagrus coronata

*Syagrus coronata* ((Mart.) Becc.) is a palm tree native to the Cerrado biome and the east coast of Brazil, also occurring in the northeast [65,123]. Vernacular nomenclature includes licuri, ouricuri (Portuguese), uricuripalme (German), licuri (Spanish), and ouricury palm and licuri palm (English) [8]. *S. coronata* palm tree size is 1.5–13 m in height, and its fruits are ellipsoid or ovoid, 2.5–3 × 1.7–2 cm, with a yellowish-green or yellowish epicarp [124].

The GBIF platform [8] reported 560 occurrences of *S. coronata* in South America (Figure 6a), specifically from northeastern (AL, BA, PE, and SE) and southeastern (MG) Brazil (Figure 6b). Cerrado and Caatinga are the main biomes where this species can be found (Figure 6c) [124,125]. It plays an essential ecological role in the Caatinga ecosystems as a source of income and livelihood for rural communities in semiarid northeastern Brazil [126]. The studies with *S. coronata* reported in the WOS are mostly related to Plant Science (21.5%), Agriculture, Dairy, and Animal Science (12.7%), and Agronomy (11.4%) [25]. On the other hand, SCO shows Agricultural and Biological Sciences (44.3%), Biochemistry, Genetics, and Molecular Biology (9.9%), and Environmental Sciences (9.9%) as the core groups [24].

Licuri bunches yield up to 1357 fruits, with an average length and diameter of 2.0 cm and 1.4 cm, respectively. The average pulp weight of a licuri fruit is 4.26 g, and the pulp to almond ratio is 86.5%. Both pulp and kernel are edible and much appreciated by local populations in Bahia, where the kernel is used to prepare “cocada,” a confectionery usually produced with coconut and sugar in northeastern Brazil [65]. Raw licuri seeds are consumed after sunlight drying or are roasted (170–190 °C/1 h) and used in regional food preparations. The oil from licuri kernels is obtained by cold press and is used for cooking and appreciated for its particular sensory profile [127]. Oliveira [128] reported that the oil from licuri kernel is used in popular medicine to treat gastrointestinal disorders in Pernambuco state.

## 5. The Oil Content in Arecaceae Palm Fruits

Fruits from Arecaceae palms are rich in oil which can be extracted from both the pulp and kernel. Table 1 presents the oil content of 13 selected Arecaceae palm fruit species. The highest lipid content was observed in *A. maripa* (68%) and *A. speciosa* (66%) kernels. On the other hand, the lowest percentages were verified in *O. bataua* (0.1%) and *O. bacaba* (0.8%) kernels. A wide variation in the oil content among each species can be noted. *A. murumuru*, *Butia*, and *S. coronata* pulps presented the lowest variations (<0.4%) on the reported oil yield, while the highest discrepancies were verified in *A. aculeata* kernel (<92%) and *O. bacaba* pulp (<75%). The main differences in the lipid content and the variability in physical characteristics and chemical composition were associated with the species, cultivation site, extraction method, and degree of fruit maturation. Detailed information on the lipid content of each Arecaceae species is discussed in the following subsections.

### 5.1. A. aculeata

The oil content in macaúba fruit varies between 10 and 29% for macaúba pulp (MAP) [28,29] and 3 and 48% for macaúba kernel (MAK) [29,30,31]. The extraction method and the solvent employed may affect the lipid recovery from these raw materials [28,29,30,31,72]. Cold pressing is the primary extraction method employed for recovering oil from macaúba fruits due to its lower cost compared to extraction using expeller or solvents. It also keeps the quality and chemical composition of the oil. Nonetheless, some studies indicated that high-quality oil could be extracted from MAP using classical methods such as Soxhlet with organic solvents *n*-hexane and dichloromethane [28] and maceration with ethyl acetate, *n*-hexane, and isopropanol [129]. Pressurized liquid extraction (PLE) with ethanol and isopropanol [130] and subcritical extraction with propane [28] have also been reported as alternative techniques to obtain MAP oil. The yield reported for MAP oil ranges from 9.77% with compressed propane (at 100 °C, 4 MPa) [28], to 19.62, 22.80, 25.64, 26.83, and 28.94% for cold press, compressed propane (at 80 °C, 8 MPa), *n*-hexane, dichloromethane, and petroleum ether (Soxhlet), respectively [28,29,31].

The lowest oil recovery of MAK (3%) was obtained by cold press extraction [31], and the highest yields were found using aqueous extraction (22–29%) [30], Soxhlet with petroleum ether (46%) [29], and ethyl ether (47.50%) [30]. Magalhães et al. [30] indicated that the aqueous extraction of MAK may be considered a feasible alternative due to the satisfactory oil yield (up to 29%) and low free fatty acids (FFA) and peroxide value (PV) when compared to the oil obtained by classic methods.

The commercialization of products made from macaúba has been increasing in Brazil through the cooperatives that help spread and disseminate their use [17]. The cold pressing oil from MAP is currently available in some specialized markets in Brazil and online stores and is sold for 78–270 USD per liter. On the other hand, extra virgin MAK oil is sold as macaúba butter for about 20 USD per kilogram.

### 5.2. A. aculeatum

The tucumã-do-Amazonas presents an oil content of 21–62% in the pulp (TAP) [32,33,34] and 28–46% in the kernel (TAK) [35]. Tucumã-do-Amazonas is one of the APTFs with the highest oil content in the pulp, which is similar to that of palm (*E. guineensis*).

Although rich in oil and representing about 39% of the seed, TAK is usually discarded as waste [35]. Depending on their locations, different physical characteristics and chemical compositions of tucumã-do-Amazonas are reported [81]. The seeds collected in the Amazonas state provided a higher yield in oil for TAK than that found in the Acre state (28–45 and 21–23%, respectively) [131]. Mechanical cold press could be a feasible option for extracting oil from TAK. Production of about 26 tons of kernel per year (dry basis) and 6–10 tons per year of TAK oil could be obtained, adding value to the coproduct currently wasted in the Amazon region [35].

The unrefined oil obtained from TAP by percolation/maceration is currently available from special online stores in Brazil and sold as tucumã butter for about 42 USD per liter.

### 5.3. A. murumuru

Murumuru pulp (MUP) yields a low oil content of 2.60% whose properties remain unrevealed [36]. On the other hand, the edible kernel (MUK), which represents about 33% of the fruit [37], is very oily and yields between 28 and 44% fat [37,38,132]. MUK is a semi-solid fat [37,38] called murumuru butter [132]. The MUK butter is described as a waxy and brittle solid fat at room temperature, with a pale-yellow color, very little odor, and no particular flavor [37,84,133]. It is obtained by cold pressing and sold in Brazilian online stores for about 46–112 USD per liter, being recommended for hair and skin hydration.

### 5.4. A. vulgare

Tucumã-do-Pará kernel (TPK) and pulp (TPP) show oil yield from 30 [37,39] to 40%, respectively [12,37,39,40,134]. TPK yields a pale-yellow oil, while TPP oil shows an intense red-orange color, indicating large amounts of carotenes [37]. Although the uses currently reported for TPP and TPK are limited, they show potential for oil production. [10,37,39,135]. In addition to cold pressing, the supercritical extraction of TPP using CO_2_ has been reported as an eco-friendly method to obtain high-quality oil [134].

### 5.5. A. maripa

The oil content in inajá fruits varies between 36 and 56% in the pulp (INP) [12,41] and 31 and 68% in the kernel (INK) [39,42,43]. An adult palm tree may produce 3.0 and 2.2 kg of pulp and kernel oils, respectively. Considering its availability in Brazil, the estimated production of inajá is 1770.5 kg of oil per hectare/year (1020.8 kg of pulp oil and 749.7 kg of kernel oil). Three hundred nineteen plants per hectare could provide about 3690.83 kg of oil per hectare/year (2134.11 kg from the pulp and 1556.72 kg from the kernel) [42].

INP oil has a yellowish to orange color [42], probably due to its high content of carotenoids [136,137]. Compressed propane and supercritical CO_2_ have been reported as alternative extraction methods to obtain oil from INP rich in β-carotene (57–89 mg 100 g^−1^ oil) [41,136]. INP oil yield obtained with different solvents and extraction methods decreases according to the following sequence: Soxhlet with methanol > Soxhlet with ethanol > pressurized ethanol > Bligh–Dyer > Soxhlet with ethyl acetate > Soxhlet with petroleum ether > Soxhlet with n-hexane > compressed propane > supercritical CO_2_ + ethanol > supercritical CO_2_ (56.2, 52.6, 47.8, 35.52, 31.1, 30.33, 28.77, 27.88, 20.4, 3.6% oil, respectively) [12,41,136].

INK represents about 8% of the fruit weight [39] and shows a low moisture content (3.77–3.85%) and high content of lipids (65% on average) [42]. The INK oil is translucid at ambient temperature, and its physicochemical characteristics suggest suitability for a wide range of products [39].

### 5.6. A. speciosa

The fruit from *A. speciosa* palm tree does not have a pulp but a hard, fibrous, and lignocellulosic shell mainly composed of fiber. It is largely used to produce charcoal and activated carbon in northeastern Brazil. The epicarp, rich in starch and fibers, is currently used as a food supplement. On the other hand, babassu kernels (BBK) show a high lipid content ranging from 50 to 66%, whose characteristics are influenced by the extraction method, including cold pressing and supercritical CO_2_ [44,45,138].

Due to its higher occurrence and availability in northeastern Brazil, BBK oil has been used for decades as a cooking oil and salad dressing in traditional dishes as an alternative to common oils such as soybean and corn oils. The oil is widely sold in non-conventional markets by small producers that use rudimentary techniques to obtain and process the oil. The online stores sell babassu virgin oil industrially produced in Brazil for about 10 USD per liter.

### 5.7. B. gasipaes

A wide variation ranging from 8 to 62% in the oil content of *B. gasipaes* pulp (PUP) was found. However, the literature reports an average oil yield between 20 and 23% for PUP [46,47,48,97]. The oil content in unripe and ripe PUP varies from 13 to 26% and 24 to 43%, respectively [48]. Peach palm kernels (PPK) represent about 9% of the fruit [39], with an oil content ranging from 12 to 24% [39,48,49]. Bereau et al. [39] reported a PUK with low acid and peroxide values and a low unsaponifiable matter content, suggesting a high-quality oil.

### 5.8. Butia spp.

*Butia* spp. pulp (BUP) has an oil content near 3% [50,51]. Studies on the extraction and characterization of oils from *Butia* kernels (BUK) are scarce because they are small, hard, and challenging to handle. On the other hand, it represents a potential oil source as it shows a high oil yield ranging from 32 to 54% [52,53].

### 5.9. E. oleraceae

The oil content in açaí pulp (ACP) ranges from 7 to 48%. Unripe açaí fruits show 7% oil, while mid-ripe and ripe fruits present 31 and 48% oil content, respectively [54]. A cultivar of *E. oleracea* called “BRS-Pará,” which was developed by researchers from EMBRAPA, produces seeds with about 20.82% oil content, mostly rich in monounsaturated fatty acids (MUFAs) (62.3%), such as oleic acid (52.1%) [139].

Ferreira et al. [140] reported the enzyme-assisted aqueous extraction (EAE) of ACP oil using commercial carbohydrases (cellulase and polygalacturonase) and pectinases (pectin lyase). An increase of 28% in the oil yield was reported for EAE compared with the Soxhlet method (from 34.91 to 47.71%). The chemical composition of açaí pulp oil was not affected by the EAE compared to organic solvent extraction such as hexane [141]. The authors suggested that the enzymatic process can be an alternative to industrial processes with organic solvents.

A few studies have been reported for obtaining oil from açaí kernel (ACK). Although it presents a low lipid content (0.2–2.8%), ACK may represent an alternative to valorize this waste because it currently has no economic value [55,56]. The oil content and sensory characteristics of ACK may vary depending on the solvent used for the extraction. A pale yellowish oil is obtained using hexane, and the yield is 0.83%. On the other hand, ethanol and methanol extractions result in yellow-greenish and reddish-brown oils with a 1.01 and 2.84% yield, respectively [55]. The oil content from white (0.22%) and purple açaí seeds (0.33%) [142], dried açaí seed flour (2.20%), and lyophilized açaí seeds (2.75%) was reported [56,143]. The cost of production and machinery versus oil yield may not be attractive for the processing of this fruit. However, considering the generation of high waste volumes from the açaí industry with no commercial value, oil extraction may be an alternative for valorizing this residue and reducing environmental impacts, contributing to sustainable processes.

### 5.10. M. flexuosa

Buriti pulp (BRP) shows an oil content ranging from 19 to 52% [12,57,58]. Buriti kernel (BRK) is often discarded after the pulp extraction or used for artisanal products, plant seedlings, and the preparation of a toasted powder called “buriti coffee” [144]. The properties of BRK and its products are not well known yet. The reported oil yield from BRK ranges between 7 and 9% [59], which is quite challenging to be extracted as the kernel is considered too hard to break. This characteristic may be the main reason for the scarcity of studies and low application in product development.

### 5.11. O. bataua

The pulp of *O. bataua* (PTP) presents an oil content ranging between 14 and 52% [12,57,60]. On the other hand, a low lipid content (from 0.06 to 1.30%) has been reported for patawa kernels (PTK) [37,61]. Such a low yield may justify the few reports dedicated to studying PTK for its oil recovery.

### 5.12. O. bacaba

The oil content in bacaba pulp (BAP), which has a very accentuated green color [37], varies from 7 to 60%, depending on the extraction method and cultivation site. For example, Canuto et al. [62] obtained 7.4% oil in BAP from Roraima state, while Cunha et al. [63] reported 44.53% oil for BAP from Pará state using Soxhlet with petroleum ether. A yield of 24.88% oil was obtained using hexane in a bench-scale extractor [37], while supercritical CO_2_ extraction yielded 45.9% oil at 60 °C and 270 bar [63] and 60.4% oil at 60 °C and 420 bar [64] in BAP samples from Pará. On the other hand, bacaba kernel (BAK) showed a lower oil content ranging between 0.83 for hexane-extracted oil from Pará samples [37] and 4.10% for petroleum-ether extracted oil from Colombia [37,61].

### 5.13. S. coronata

The oil content in licuri pulp (LIP) ranges from 4.11 to 4.50% [65,66,67], while the oil from licuri kernel (LIK) ranges between 39.0 and 50.0% [65,66,68]. The physicochemical properties, fatty acid composition, and other chemical characteristics of LIP have not been reported in the literature yet. LIK is an important food and feed resource for humans and animals in semi-arid zones. In northeastern Brazil, the local populations widely use it for frying, cooking, and soap production [145].

## 6. Characteristics of Arecaceae Palm Tree Fruit Oils (APTFOs)

### 6.1. Physicochemical Properties

The APTFOs have different physicochemical characteristics depending on the part used for their extraction (Table 2).

These identity and quality parameters usually indicate the suitability for industrial applications of the oils. As can be seen in Table 2, many of these parameters for APTFOs have not been reported yet in the literature. The survey conducted in this study revealed that most differences in the physicochemical properties from the same genus and specie of APTFOs were mainly attributed to the harvesting location, the initial quality of the raw materials, the extraction method, and the storage conditions.

Most of the APTFOs showed low concentrations (<6%) of free fatty acids (FFAs), indicating the good quality of the extracted oils. On the other hand, BAP and BAK showed FFAs ranging from 2.4 to 63.0% and 35.6%, respectively. FFA and acid value (AV), which measure triglyceride’s hydrolysis products, represent important quality indices for oils. FFA and AV in vegetable oils are associated with handling and storing the raw materials. Freshly extracted oils from fresh raw materials harvested and stored in proper conditions of temperature and humidity usually present low FFA and/or AV concentrations. Conversely, oils with high levels of FFAs indicate the use of raw materials with low quality and/or failures in the steps before processing, such as harvesting, preparation, and/or storage of raw materials. The content of FFAs is reported as a percentage related to the major fatty acid in the lipid source. Due to the high occurrence of oleic acid in vegetable oils, most results are based on this fatty acid. However, FFA calculation is based on the lauric acid content (C12:0) for kernel oils since they usually present a significant content of this fatty acid.

The AV ranges from 0.2 to 20 mg KOH g^−1^ for most APTFOs. However, high acidity values were reported for ACK oil (93.1 mg KOH g^−1^) obtained by Soxhlet with hexane [55] and MAK oil (112 mg KOH g^−1^) obtained commercially [68]. Okada et al. [55] explain that such high AVs may be associated with lipases in the seeds, which can hydrolyze the triacylglycerols and increase FFA content before the oil extraction. Iha et al. [68] also suggest that different degrees of maturation and poor conditions in the storage of raw materials may impact the AV of oils from palm species.

TAP oil presented a higher acidity value (19–20 mg KOH g^−1^) compared to TAK oil (0.26–0.48 mg KOH g^−1^) [35,134]. Tilahun et al. [72] reported that the post-harvest practices directly impact the quality of the MAP and its physicochemical properties depending on storage conditions. They indicated that the MAP oil presented a good quality when the fruits were stored for 20 days at room temperature (23 ± 1 °C). High FFA and AVs are related to the lack of good practices during harvesting and manufacturing, transportation and storage, long-term storage, and high-temperature exposure before the extraction. In addition to the good practices for obtaining oil with a low FFA content and high stability, the quality can also be improved by using gamma irradiation in the fruits [146].

Most APTFOs present a low peroxide value (PV) ranging between 0.2 and 10 mEq O_2_ kg^−1^. These values are found in fresh oils and suggest oxidative stability. Nevertheless, MUK, INP, PUK, ACK, PTK, and BAP oil presented high a PV, indicating that those oils may have undergone oxidative processes, either chemical or enzymatic. Oils with peroxide values higher than 15 mEq O_2_ kg^−1^ have a low quality, indicating a high degree of deterioration.

APTFOs show iodine values (IV) ranging from 51 to 81 g I_2_ 100 g^−1^, indicating a high degree of unsaturated fatty acids (UFAs). This parameter indicates that pulp oils are liquid at ambient temperature. On the other hand, the oil obtained from the TAP shows a very low IV (10.88 g I_2_ 100 g^−1^), suggesting that it is solid at room temperature. However, other studies reported a high content of UFA for TAP oil [34,134], indicating a liquid appearance. Conversely, the kernel oils show a higher IV, suggesting a composition rich in SFA and a solid or semi-solid consistency at ambient temperature [32].

A higher saponification value (SV) was observed in kernel oils compared to pulp oils, indicating FAs with lower molecular weights in their structure. APTFOs show a refraction index (RI) between 1.43 and 1.47, which is related to their FA composition rich in SFAs, low IV, and higher protection against oxidation.

The unsaponifiable matter (UM) of 11 oils ranged between 0.11 and 3.0%, indicating a great variability in nonglyceride compounds in APTFOs. The oils with the highest range of UM are those from TPP, BAP, and PUK, showing 2.2, 2.6, and 3.0% of UM, respectively. The UM in a lipid matrix includes lignans, tocopherols, tocotrienols, triterpene alcohols, sterols, and squalene. The presence of UM may affect properties such as the conservation and stability of the oil [155]. In the diet, the compounds present in UM are related to different bioactivities, such as anti-inflammatory and cholesterol-lowering properties [155].

The oxidative stability index (OSI) is obtained using an accelerated oxidation method at high temperatures to simulate storage conditions and predict shelf life. The OSI determined at 110 °C for APTFOs varies from 0.07 to 69.9 h. The highest OSI values were observed for MUK and LIK oils (40.0 and 69.6 h, respectively). These results are related to the high SFA concentration observed in those oils. On the other hand, the high oxidative stability of 37.2 ± 2.4 h reported for BRP [19] is comparable to that observed for vegetable fat obtained from cupuassu (*Theobroma grandiflorum*) seeds [156]. Low OSI values for oils such as those from MAK, TPK, BBK, and PTK may be related to low-quality raw materials and poor manufacturing practices. As the minimum OSI required for biodiesel production is 6 h [72], many APTFOs could be suitable for such a purpose.

Other factors such as the extraction technique also affect the thermal stability of APTFOs such as BBK oil. Bauer et al. [138] evaluated the thermal degradation of extra virgin and virgin BBK oils by thermogravimetry and showed high stability at 165 and 125 °C, respectively. Such oils are usually extracted by roasting and crushing the kernel or using raw kernels in a cold mechanical press. Although presenting slight differences in the chemical profile, exposing the kernels to high temperatures during roasting may affect the content of minor compounds that have a critical role in the stability of vegetable oils. However, small producers and consumers prefer using roasted kernels due to the characteristic flavor that the oil presents.

The melting point (MP) of the APTFOs presents a wide variation (between 8.0 and 37.2 °C), indicating different FA compositions. Oils with high UFAs usually show a low MP and appear as liquid oils at ambient temperatures. On the other hand, the oils rich in SFAs are solid or semi-solid at ambient temperature. The MP for most oils from APTFs has not been reported yet.

The reported kinematic viscosity (KV) and density (ρ) of APTFOs range between 15.4 and 44.2 mm^2^ s^−1^ and between 860 and 952 kg m^−3^, respectively. The KV and ρ are usually measured at 40 °C to ensure a complete liquid state for the fat or oil. However, some authors report ρ at 20 °C, which makes the comparison among samples difficult. These temperature-dependent properties affect the oils’ flowing properties and rheological behavior [157,158]. Thus, the development, dimensioning, and specification of machinery, reactors, and unit processes such as pumping, heat exchanging, and piping should consider such properties. Oils with a high viscosity and density may solidify in equipment and clog pumps, requiring jacketed and heated systems to facilitate pumping in industry. The lower these values, the easier it is to mix and pump the oils in industry.

### 6.2. Fatty Acids

The lipid composition of palm fruit pulps has remarkable differences from that of the kernel. Pulp oils are richer in UFAs, while kernel oils have a higher degree of saturated fatty acids (SFAs). Table 3 shows that among the 25 APTFOs, only MUP and BAK have not had their fatty acid (FA) profiles investigated so far.

The SFAs content in the kernel oils varies from 11 to 98%. MAK, TAK, MUK, TPK, INK, BBK, PUK, BUK, and LIK oils are rich in SFAs, with more than 85% SFAs in their composition. On the other hand, ACK, BRK, and PTK oils present less than 34% of SFAs. The kernel oils present between 5 and 87% of monounsaturated fatty acids (MUFAs) and 2–49% of polyunsaturated fatty acids (PUFAs). On the other hand, in pulp oils, MUFAs, PUFAs, and SFAs account for 39–80%, 1–20%, and 13–47%, respectively. BRP and PTP oils are the major sources of MUFAs, whose content ranges between 72 and 80%. BRK oil presents the highest concentration of PUFAs with up to 49%, mainly linoleic (16–36%) and linolenic (2–13%) acids.

MAP oil is mostly composed of MUFAs (55–68%). Oleic (55–63%) and palmitic (23–30%) acids are the main FAs found in this raw material. Linoleic (5–10%) and linolenic (1–3%) acids were also reported [28,31]. On the other hand, MAK oil is rich in SFAs (55–89%), mainly lauric (32–46%), palmitic (7–11%), and myristic (8–12%) acids. Minor contents of stearic (2–6%), caprylic (1–6%), capric (~4%), and linoleic (~3%) acids were reported in MAK oil as well [30,31,162].

TAP oil has a FA composition majorly based on MUFAs and PUFAs such as oleic (64–74%) and linoleic (11–12%) acids, respectively. The main SFAs are palmitic (8–10%) and stearic (5–7%) acids [34,134]. Differently, TAK oil displays a FA composition rich in SFAs (86–98%), composed mainly of lauric (52–58%) and myristic (23–27%) acids. Palmitic, stearic, oleic, and linoleic acids content ranges between 2 and 7%. Minor caprylic and capric acid contents (<2%) were also reported.

No report has been found on the FA composition of MUP oil. MUK butter has a FA profile composed mostly of SFAs (84–95%), such as lauric (47–52%), myristic (26–29%), and palmitic (6–7%) acids. MUFAs and PUFAs account for 7–8% and 3–4%, respectively. The major FA found in MUK butter is lauric acid, whose content ranges between 47 and 52%, followed by myristic acid (26–29%). Oleic acid is reported in minor proportions (6–8%). Other FAs include linoleic, stearic (~3%), caprylic, capric (1–2%), arachidic, and linolenic (<0.1%) acids [10,11,15,37].

The FA profile of TPP oil, which is rich in oleic acid (65–68%), has been evaluated by several authors [12,85,97,134,164,165,166,167]. The SFAs are composed of palmitic (21–25%), stearic (2.6–5.1%), and arachidic (4%) acids. The oil presents a low amount of PUFAs such as linoleic (<5%) and linolenic (<3%) [12,134,148,159]. Other FAs reported in small concentrations (<1%) include tricosanoic, myristic, lauric, arachidonic, gondoic, docosadienoic, margaric, palmitoleic, lignoceric, and behenic acids [160]. Costa et al. [134] reported that temperature is an important parameter for extracting TPP oil using supercritical CO_2_. The temperature (50–60 °C) has a significant impact on the FA composition of TPP oil. The highest content of oleic acid was obtained at 50 °C. Conversely, the authors observed a higher concentration of linoleic acid at 40 °C. The FA composition of TPK oil includes mainly SFAs (75–94%) such as lauric (44–51%), myristic (25–29%), and palmitic (6–7%) acids. The UFAs in TPK oil are composed mostly of oleic (8–14%) and linoleic (~3%) acids [39,44]. Other FAs such as capric, caprylic, and stearic (<2%) were found in TPK as well [44].

INP oil is majorly composed of MUFAs (39–62%), mainly oleic acid (up to 61%). The oil also presents a high content of PUFAs (7–19%), mostly linoleic acid (6–13%). The content of SFAs (24 to 47%) is mostly composed of palmitic (20–25%), myristic (2–10%), and stearic (<3%) acids. In contrast, INK oil has a high content of SFAs (35–97%). This oil can be classified as lauric since the concentration of lauric acid is up to 40% of its FAs. Other SFAs include myristic (14–25%), palmitic (9–21%), and stearic (<2%) acids. The UFAs found in INK oil are mainly oleic (11–43%), linolenic (~8%), and linoleic (2–7%) acids [39,42]. Although presenting a high potential for oil production, the properties and chemical composition of INK are not well known, and detailed information on its characteristics is scarce in the literature.

BBK oil is mainly composed of SFAs (84 to 95%), mainly lauric (48–50%), myristic (~14%), caprylic (~8%), capric (~7%), and palmitic (~7%) acids. MUFAs represent around 9% of its composition and minor contents of stearic (~3%), undecylic (<2%), tridecylic (<1.5%), linoleic, linolenic (<1.6%), caproic (<0.5%), and palmitoleic (<0.2%) acids can also be found [11,138,168]. Margaric and heneicosylic acids have been reported as traces (<0.1%). The FA profile of BBK oil is similar to that of palm and coconut oils [138], representing an alternative to those commodities.

PUP oil is a rich source of MUFAs (47–68%), mainly oleic (36–61%) acid. High contents of SFAs (25–51%), mainly palmitic acid (24–51%), have been found. Other FAs such as palmitoleic (3–7%), linoleic (2–5%), stearic (0.8–3.5%), and linolenic (~1%) acids were reported as well. Margaric and myristic acids in PUP oil were found as traces (<0.1%) [46,47,148]. The FA profile of PUK oil presents a large variability depending on the cultivation country. PUK oil from Brazilian yields is rich in lauric (60.6%) and myristic (18.9%) acids, while that from Ecuador was found to have a balanced composition of lauric (33.3%), myristic (27.8%), and oleic (24.3%) acids [47,49]. PUK oil is mainly composed of SFAs (76–86%), and the only unsaturated FA identified was oleic acid (13%). Other FAs such as caproic, caprylic, capric, stearic, and linoleic acids were found as traces [39].

Data on the FA composition of both oils from the pulp (BUP) and kernel (BUK) of *Butia* species are still scarce in the literature. Lopes et al. [51] reported contents of ~48% MUFAs, 35–38% SFAs, and 14–15% PUFAs in BUP oil from *B. capitata*. The main FAs were oleic (47–48%), palmitic (~19%), linoleic (11–12%), and caproic (~7%) acids. On the other hand, Faria et al. [52] reported a high content of lauric acid (42%), followed by oleic (17%), myristic (11%), capric (8%), caprylic (8%), palmitic (6%), and stearic (4%) acids for BUK oil from *B. capitata*. Linoleic acid has also been reported at 4%.

ACP oil is mainly composed of oleic acid (52–62%) and palmitic acid (23–27%). ACP oil also presents linoleic (7–11%), palmitoleic (4–5%), and stearic (<2%) acids [139,141,159]. A concentration between 3.39 and 4.80% of *cis*-11-octadecenoic acid (or *cis*-vaccenic acid, C18:1*n*11) has been reported. This C18:1 isomer is an omega-7 usually found in high concentrations in fruit pulps such as Japanese persimmon (*Diospyros kaki*), Japanese mandarin (*Citrus unshiu*), grapefruit, sweet orange, and mango (29.0, 22.3, 19.4, 16.9, and 14.3%, respectively) [169,170]. ACP also presents lower than 1% linolenic, arachidic, myristic, and lauric acid contents [139,141].

Studies on the extraction, physicochemical and chemical composition, and application of ACK oil are still limited. Most research using açaí seeds focuses on its use as a raw material for biofuel production, construction material, activated carbon, and fiber extraction. The oil yield for ACK is low, and the hardness of the seed makes it challenging to obtain and characterize it. The ACK oil mainly comprises triglycerides and FAs (99.1%). The diglycerides, sterols, and gum substances represent 0.27, 0.40, and 0.22%, respectively. The FA profile of ACK oil indicates an oil rich in MUFAs (53%) and PUFAs (15%). The oleic (27–51%), linoleic (15–26%), myristic (5–23%), and palmitic (~16%) acids are the major FAs in ACK oil. It also has lauric (3–9%), stearic (1–3%), arachidic (2%), and gondoic (1.5%) acids, as well as traces of palmitoleic acid [55,56].

BRP oil is rich in MUFAs (72–80%). SFAs and PUFAs account for 20–24% and 1–4%, respectively [11,117]. Oleic acid (72–79%) was described as the major FA found in BRP oil, followed by palmitic acid (18–21%). It also presents other FAs, such as stearic (<1.6%) and linoleic (1–2%), and low contents (<1%) of arachidic, palmitoleic, myristic, linolenic, margaric, pentadecanoic, and lauric acids [11,12,148]. On the other hand, BRK oil is mainly composed of PUFAs (28–53%) and MUFAs (18–49%). SFAs in BRK oil account for 22–30%. The main FAs in BRK oil are oleic (28–53%), linoleic (16–36%), palmitic (18–24%), and stearic (4–5%) acids. A substantial content of linolenic acid varying from 2 to 13% has been reported as well [59].

Oleic and palmitic acids are the major FAs in PTP oil [10,11,12,57,159]. The oleic acid content in PTP oil can vary depending on the country. The literature indicates oleic acid contents of 82.53%, 81,91%, 77.7%, and 46.06% for samples from Colombia, Peru/French Guyana, Brazil, and Venezuela, respectively. These variations on the FA profile of *O. bataua* may be due to misidentification, which also happens to *O. bacaba*, or sampling with hybrids [60]. Most reports showed that PTP oil mainly comprises UFAs (81–82%). About 78–80% MUFAs and 4–5% PUFAs have been reported in PTP oil. Oleic acid accounts for 78–79%, followed by 11–12% palmitic acid. The PTP oil also presents low contents of linoleic (4–5%) and stearic (3–4%) acids, and <2% of lauric, arachidic, linolenic, myristic, palmitoleic, pentadecanoic, caprylic, and capric acids [10,11,159]. Similarly, PTK oil is mainly composed of MUFAs (66–87%), SFAs (11–35%), and a minor content of PUFAs (1–7%). The main fatty acids in PTK oil are oleic (65–82%) and palmitic (10–26%) acids. Lower contents (<1%) of arachidic, gondoic, margaric, heptadecenoic, pentadecanoic, myristic, tridecanoic, lauric, caproic, and capric acids were also reported in PTK oil [10,11,12,37,57,159].

BAP oil is mainly composed of MUFAS (45–67%) and SFAs (20–31%) with a significant content of PUFAs (13–21%). The composition of BAP oil is primarily oleic acid (46–66%), followed by palmitic (17–26%), linoleic (12–20%), and stearic (2–5%) acids. Low contents (<1%) of palmitoleic, myristic, linolenic, arachidic, and behenic were also reported [63,148]. The high oxidative stability reported for BAP oil has been ascribed to its rich FA composition [37,64,148,152]. Studies on the composition of BAK were not found in the literature so far.

Silva et al. [161] reported the identification of only three FAs in LIP oil: palmitic (42%), stearic (32%), and oleic (25%) acids. On the other hand, the FA composition of LIK oil has been widely studied [68,153,154,163,171]. LIK oil is a rich source of SFAs (72–98%), mainly lauric (36–48%), myristic (14–16%), and caprylic (9–13%) acids. Other FAs in LIK oil include oleic (7–12%), palmitic (5–9%), capric (6–8%), linoleic (~3%), and stearic (2–4%) acids [68,153,154,163].

### 6.3. Triacylglycerols

Table 4 shows that 43% of the 25 APTFOs discussed herein had their TAG profile reported. Pereira et al. [10] reported the presence of LLM (15%), LLL (14%), MML (8%), LLP (7%), and PLM (7%) for MUK oil. These results are in agreement with the FA composition rich in lauric, myristic, and palmitic acid described by other authors [10,11,37]. The TPP oil is mainly composed of POO (39%), OOO (19%), and POP (22%) [148], which was confirmed by the FA profiles reported for this oil [12,148,159,160]. TPK oil is composed of LLM (23%), LLL (20%), MML (15%), PLM (7.5%) and LLO (6%) as the main TAGs [10]. About 24 different TAGs have been identified in INP oil, mainly POO (13%), POP (12%), MOP (10%), and MPP (8%) [148].

Bauer et al. [138] reported 26 TAGs in BBK oil, mainly LLL (14–15%), LOL (11–13%), LLM (10–11%), LLC (8–9%), LLCp (~8%), LLP (~7%), and LOM (~6%) [138].

Santos et al. [148] reported POP (32%), POO (29%), and POPo (12%) as the main TAGs in PUP oil, correlated with the rich composition in palmitic and oleic acids [46,47,148]. Sixteen TAGs, mainly POO (25–39%), OOO (29–26%), and OOL (11%) [148,172], were reported for BRP oil, corresponding to the high content of oleic and palmitic acids [11,12,148].

Only four TAGs were reported for PTP oil such as OOO (39%), OOP (19%), OOL (9.5%), and OOS (8%) [10]. Santos et al. [148] reported 14 different TAGs for BAP oil, mainly POO (19%), POL (18%), POP (10%), and OOO (9%) [63,148].

The major TAGs found in LIK oil were CpLL (26%), CLL (21%), CLM (15%), and LLM (12%) [69], which are in line with its high content of lauric, myristic acids, caprylic, and capric acids [68,153,154,163].

The TAG profile of oils and fats has a major role when considering their industrial application. The position of the FAs in the glycerol backbone drives some physical and chemical properties such as melting point and oxidative stability. In addition, it is also very important from a nutritional point of view since the position of the FAs in the TAG can change their absorption in the human body.

### 6.4. Bioactive Compounds

Table 5 shows the main bioactive compounds reported in APTFOs. Palm fruit pulps are rich in bioactive compounds such as carotenoids [28,29,39,43,72,136,173]. The literature reports the content of bioactive compounds for 58% of the 25 oils shown in this review. Most of the studies have assessed the content of tocopherols, but only a few reports investigated the presence of other lipids such as carotenoids and phytosterols. APTFOs are rich in tocopherols, mainly α-tocopherol, which has vitamin E activity. PTP and BRP oils present the highest concentration of tocopherols (up to 1125 and 1704 mg kg^−1^ oil of α-tocopherol, respectively). Other oils such as those from MAP, MUK, BBK, PTK, and TPP also presented a high content of α-tocopherol. As a result of the high content of tocopherols, some oils have significant α-tocopherol equivalent activity (α-TE), usually correlated with the antioxidant capacity in the human body. The highest α-TE was registered for the oils of BRP (842.30 α-TE 100 g^−1^), MUK (136.88 α-TE 100 g^−1^) [11], and PTK (59.1 α-TE 100 g^−1^) [12]. Low levels of tocotrienols (mostly α-tocotrienol) in APTFOs have been reported, ranging between 1 and 91 mg kg^−1^ oil for PUK and BRP oils, respectively.

The yellowish color reported for APTFOs has been associated with the presence of carotenoids [12,29,37,173,174]. The highest concentration of carotenoids, from 130 to 3560 mg kg^−1^ oil, has been reported for MAP [28,29,72], INP [12,43,174], TPP [12,37,174], and BRP [11,12,137,172,174] oils. The main carotenoid in these raw materials is β-carotene (3.0–3.560 mg kg^−1^ oil). However, data on the content and/or profile of carotenoids for more than 60% of the APTFOs described in this study have not been reported in the literature yet.

TPP is rich in carotenoids (between 135.5 and 163.7 mg kg^−1^) [164,175] where all-*trans*-β-carotene represents 212.69 μg g^−1^ [149]. The peels of *A. vulgare* are also rich in carotenoids such as all-*E*-β-carotene (7.8 mg 100 g^−1^) and all-*E*-γ-carotene and *Z*-γ-carotene (2.6 and 0.8 mg 100 g^−1^, respectively) [176]. The TPP oil has been reported as the highest source of carotenoid among the APTFOs already evaluated (1222–2420 mg kg^−1^ oil). The main carotenoids reported in TPP oil were β-carotene (567.08 mg kg^−1^) and *cis*-β-carotene (230.92 mg kg^−1^), followed by neoxanthin, *cis-*γ-carotene, γ-carotene, and lutein (76.08, 75.87, 68.02, and 44.34 mg kg^−1^ oil, respectively). Other carotenoids such as α-carotene, 5,8-epoxy-β-carotene, luteoxanthin, β-cryptoxanthin, violaxanthin, and *cis-*violaxanthin were reported in TPP in a range between 12.80 and 29.21 mg kg^−1^ oil [137].

**Table 5:** Bioactive lipids of Arecaceae palm fruit oils.

Compound	MAP	MAK	MUK	TPP	TPK	INP	INK	BBK	PUP	PUK	BRP	BRK	PTP	PTK	BAP	LIP
	Tocopherols (mg kg^−1^ oil)															
**α-Tocopherol**	17.1–143.70	14.25	89.94	52.0	7–16	20.0–114.85	2–3	42.16	47	2–3	252.1–1125.0	nd	1704	56.5	-	-
**β-Tocopherol**	3.25	0.85	91.52	-	-	25	1	nd	7	-	71.3–761.87	-	-	7.8	-	-
**γ-Tocopherol**	57.85–145.7	nd	10.09	-	-	50.97	1	11.91	4	-	56.71–1074.0	616.9	269	-	-	-
**δ-Tocopherol**	6.7–24.7	7.90	5.65	nd	-	nd	tr	12.09	-	-	44.1–224.18	378.82	-	7.7	-	-
**α-Tocotrienol**	-	-	nd	-	55–59	36	6–7	nd	-	1–2	90.80		-	-	-	-
**β-Tocotrienol**	-	-	nd	-	-	12	2	nd	-	-	nd		-	-	-	-
**γ-Tocotrienol**	-	-	nd	-	-	10	2	nd	-	-	nd		-	-	-	-
**δ-Tocotrienol**	-	-	nd	-	-	10	tr	nd	-	-	10.60		-	-	-	-
**Total tocopherols**	126.9–212.95	23.10	197.2	-	12–18	26.6–185	5–15	-	59	90–115	1129.78	995.72	-	72.0	-	-
α-TE *	-	-	136.88	52.9	-	22.0	-	43.71	-	-	56.00–842.30	73.32	-	59.1	-	-
**Total vitamin E**	-	-	197.2	-	-	-	-	66.16	-	-	1511.01		-	-	-	-
	**Carotenoids (mg kg^−1^ oil)**															
**β-Carotene**	1366.6–3560.5	-	-	-	-	571.4–1409.9	-	-	150.19	-	295.24–781.6	-	2.38	-	3.02	-
**Total carotenes**	130–300	1.82	-	1222–2420	3	-	-	-	357.42	-	540–1722.87	-	22	-	13.53	87.0
	**Phytosterols (mg kg^−1^ oil)**															
**Brassicasterol**	-	-	-	-	-	3.50	-	-	-	-	2.50	nd				
**Campesterol**	28–338.7	-	-	13.9–133.2	84–87	18.8	-	-	6.3–10.9	-	6.6–16.0	8.0	7.2	-	11.0	-
**Campestanol**	-	-	-	-	-	-	-	-	-	-	-	-	6.0	-	-	-
**Stigmasterol**	48.1–152.6	-	-	8.1–66.1	35–40	5.4	-	-	2.9–4.2	-	16.8–38.5	6.0	19.2	-	12.6	-
**Δ5,23-Stigmastadienol**	-	-	-	-	-	4.1	-	-	-	-	-	-	-	-	-	-
**β-Sitosterol**	173.1–1496.5	-	-	76.6–488.2	1353–1363	65.4	-	-	51–3-55.0	-	76.6	6.0 **	34.2	-	76.4	-
**∆5-Avenasterol**	-	-	-	1.4	638–648	2.4	-	-	2.7–4.8	-	-	-	27.8	-	-	-
**Δ5,24-Stigmastadienol**	-	-	-	-	-	2.3	-	-	-	-	-	-	-	-	-	-
**Δ7-Stigmastenol**	-	-	-	-	-	0.6	-	-	-	-	-	-		-	-	-
**Squalene**	-		-	58.8	-		-	-	16.9	-	-	-	-	-	-	-
**Total phytosterols**	233.9–1881.7	-	0.8	1497.2–2708	1.1	1463	-	-	4456	-	2332	-	0.7–368	-	981	-
**References**	[28,29,72]	[29]	[11,37]	[12,37,164,174]	[37,39]	[12,43,174,177]	[39,43]	[11]	[49,174]	[39]	[11,12,137,172,174,177]	[177]	[37,60]	[12]	[37,137,174]	[161]

* α-Tocopherol equivalent activity (α-TE 100 g^−1^); ** β-sitosterol + sitostanol; nd, not detected; tr, trace. Acronyms: MAP and MAK, macaúba pulp and kernel oils (*A. aculeata*); MUK, murumuru kernel butter (*A. murumuru*); TPP and TPK, tucumã-do-Pará pulp and kernel oils (*A. vulgare*); INP and INK, inajá pulp and kernel oils (*A. maripa*); BBK, babassu kernel oil (*A. speciosa*); PUP and PUK, peach palm pulp and kernel oils (*B. gasipaes*); BRP and BRK, buriti pulp and kernel oils (*M. flexuosa*); PTP and PTK, patawa pulp and kernel oils (*O. bataua*); BAP, bacaba pulp oil (*O. bacaba*); LIP, licuri pulp oil (*S. coronata*).

INP showed high carotenoid content (85.03 mg kg^−1^ oil), mainly β-carotene, cis-lycopene, lycopene, γ-carotene, cis-γ-carotene, and cis-β-carotene (23.03, 15.49, 15.33, 14–85, 9.81, and 6.01 mg kg^−1^ oil, respectively) [137]. Compressed propane and supercritical CO_2_ were reported as good solvents to obtain INP rich in bioactive compounds such as carotene, fumaric, ascorbic, vaccenic acids, and β-carotene [136].

A high carotenoid content has also been found in PUP oil (357.42 mg kg^−1^ oil), mainly β-carotene and γ-carotene (150.19 and 67.62 mg kg^−1^ oil, respectively). The oil also shows *cis*-γ-carotene, lycopene, *cis*-β-carotene, *cis-*lycopene, and lutein (35.40, 30.80, 27.66, 26.84, and 11.94 mg kg^−1^ oil, respectively), and minor contents (<3 mg kg^−1^ oil) of δ-carotene, *cis*-lutein, and *cis-*δ-carotene [137]. Radice et al. [49] reported 59 mg kg^−1^ oil for the total tocopherol content in PUP oil from Ecuador. The main tocopherol identified was α-tocopherol (47 mg kg^−1^ oil), followed by minor contents of β- and γ-tocopherols (7 and 4 mg kg^−1^ oil, respectively).

APTFOs present phytosterols as well. High content of phytosterols has been reported for PUP, TPP, and BRP oils. The reported oils can be categorized regarding the content of phytosterols as follows: PUP > TPP > BRP > MAP > INP > BAP > TPK > MUK > PTP. Studies on the phytosterols have not been reported yet for most APTFOs.

TPP oil presents a significant content of other phytosterols such as arundoin (241.9 mg kg ^−1^) and cycloartenol (170.3 mg kg^−1^). The literature has reported anti-inflammatory properties of TPP [164] associated with its carotenoids, phytosterols, and tocopherols content. Bony et al. [167] suggested that the consumption of tucumã fruit can prevent several inflammatory diseases. Del Río et al. [31] reported that MAP oil is richer in sterols than MAK oil (1.50 and 0.21%, respectively). Similarly to TPP, the PTP presents a high content of cycloartenol (105 mg kg^−1^) [60]. In addition to a rich nutritional composition, the peels and pulp from *A. aculeatum* have a high bioactive compound content, mainly β-carotene and quercetin. These compounds have been associated with cytoprotective effects on human lymphocyte cells under H_2_O_2_ [178].

Açaí has been widely reported as a superfruit due to its high content of bioactive compounds, such as anthocyanins (2.63 mg g^−1^) [179], orientin (8.05 mg g^−1^), apigenin (3.49 mg g^−1^), *p*-coumaric acid (3.52 mg g^−1^), cyanidin-3-*O*-glucoside (2.62 mg g^−1^), luteolin (2.57 mg g^−1^), epicatechin (2.37 mg g^−1^), and vixetin (2.19 mg g^−1^) [180]. Higher contents of biologically active compounds such as vanillic, syringic, *p*-hydroxybenzoic, and protocatechuic acids have been reported for ACP oil (1610–1616, 969–1072, 570–892, and 540–630 mg L^−1^, respectively) when compared to pulp extract (577, 434, 172, and 159 mg L^−1^, respectively) obtained with ethyl acetate [181,182]. Bioactive compounds in ACP oil include flavan-3-ols such as procyanidin trimers and dimers, and (+)-catechin (2016–2040, 1030–1086, and 67 mg L^−1^, respectively). The high thermal stability when stored at <40 °C for ten weeks and heated to up to 170 °C for 20 min was associated with these antioxidants [181]. The ACP oil showed higher activity (2.1-fold) as an inhibitor against the proliferation of HT-29 human colon adenocarcinoma cells compared to the pulp extract obtained with ethyl acetate [182]. The cytotoxicity of ACP oil in rat liver and thyroid tissues [183] and its genotoxicity in mammalian cells in vivo were evaluated [184]. The results indicated that ACP oil did not present genotoxic effects in the animals’ leukocytes, liver, bone marrow, and testicular cells. However, damage was observed in cells and tissues of the liver and thyroid, suggesting that the oil may present cytotoxic activity.

Okada et al. [55] showed a total phenolic content of 0.6212 mg GAE mL^−1^ and total flavonoids of 0.3587 mg CE mL^−1^ in methanolic extracts of ACK oil. The oil also presented radical scavenging activity between 0.3 and 87.1% (DPPH assay). The chemical profile of ACK oil indicates that it may have high oxidative stability and may stimulate its extraction to add value to açaí residues. The INP is another raw material rich in phenolics (989.6 to 1711.3 mg GAE g^−1^) [185] and flavonoids (34.14 mg 100 g^−1^) [166].

The total carotenoid content reported for BRP ranges between 17.00 and 52.86 mg 100 g^−1^ [116,117]. BRP is also a rich source of tocopherols (800 to 1129.78 μg g^−1^) [117,177]. The main tocopherol in BRP is γ-tocopherol (878.45 μg g^−1^), followed by α- and δ-tocopherols (252.15 and 224.18 μg g^−1^, respectively). The α-tocopherol equivalent (α-TE) observed in BRP (346.72) is considered one of the highest reported when compared to other fruits and nuts such as uxi (*Endopleura uchi*) pulp (200.92), red açaí pulp (147.72), INP (117.39), and Brazil nut (80.16). BRK also has a high total tocopherols content (995.72 μg g^−1^), composed of γ- and δ-tocopherols (616.90 and 378.82 μg g^−1^, respectively) [177]. BRP is also a rich source of phytosterols (183.0–265.0 mg 100 g^−1^). The major phytosterols reported in BRP were β-sitosterol + sitostanol (154.50 mg 100 g^−1^), followed by stigmasterol, campesterol, Δ^5^-avenasterol + Δ^7^-stigmasterol, and brassicasterol (38.50, 16.0, 3.50, and 2.50 mg 100 g^−1^, respectively). On the other hand, a 7–10-fold lower content of total phytosterols (25.0 mg 100 g^−1^) has been reported for BRK. In BRK, the phytosterols are mainly composed of campesterol, stigmasterol, β-sitosterol + sitostanol, and Δ^5^-avenasterol + Δ^7^-stigmasterol (8.0, 6.0, 6.0, and 5.0 mg 100 g^−1^, respectively) [177]. Part of such compounds is recovered in oils, as the lipid phase presents many bioactive compounds [11,12,137,172,174].

BRP oil has been reported as one of the highest carotenoid sources (540.81 mg kg^−1^ oil) among APTFOs. The main carotenoid is β-carotene (295.24 mg kg^−1^ oil) and *cis-*β-carotene (165.65 mg kg^−1^ oil), followed by lutein, α-carotene, *cis*-lutein, 5.8-epoxy-β-carotene, γ-carotene, luteoxanthin, *cis*-γ-carotene, and cis-α-carotene (32.12, 19.20, 16.28, 4.38, 3.45, 2.68, 1.82, and 1.80 mg kg^−1^ oil, respectively) [137]. The sterols present in PTP oil are β-sitosterol (34.2%), Δ^5^-avenasterol (27.8%), stigmasterol (19.2%), campesterol (7.2%), campestanol (6.0%), and cholesterol (3.4%).

BAP oil has low carotenoid content (13.53 mg kg^−1^ oil) compared to other palm fruits. The main carotenoids reported for BAP oil are lutein, β-carotene, *cis*-lutein, and α-carotene (6.20, 3.02, 1.78, and 1.05 mg kg^−1^ oil, respectively) [137]. LIK oil showed activity against *Staphylococcus,* and it was related to its high content of lauric and oleic FAs [186]. The FAs from LIK also showed larvicidal and oviposition-deterrent activities against *Aedes aegypti* [187].

The literature indicates that PTP has phenolic compounds such as piceatannol [120], a stilbene analog to resveratrol primarily found in red fruit grapes. Piceatannol has been associated with antioxidant, antilipogenic, and anti-inflammatory activities in human and animal models [188].

The yellowish LIP is composed of β-carotene and α-tocopherol (26.1 and 3.8 mg kg^−1^, respectively) [65]. Belviso et al. [127] showed that roasting significantly improves the content of bioactive compounds and the antioxidant activity in LIK. Total phenolics, flavonoids, and condensed tannins content increased from 1.21 mg GAE g^−1^, 1.16, and 1.17 mg catechin g^−1^, respectively, to 2.78 mg GAE g^−1^, 2.12, and 2.02 mg catechin g^−1^ after roasting. The authors also revealed that the content of individual phenolics such as procyanidin B1 and B2, catechin, epicatechin, and quercetin increase after roasting processes, enhancing their nutritional value. Consequently, the ABTS and DPPH radical scavenging activities were improved, respectively, from 4.07 and 5.88 to 5.31 and 7.01 mmol TE g^−1^ [127]. The authors suggested that roasting may enhance the content of bioactive compounds, probably due to the release of phenolics from bounded forms, thus improving the quality properties of LIK.

### 6.5. Similarities in the Lipid Composition among APTFOs

Table 3 shows that the UFAs are the main FAs in pulp oils. On the other hand, the SFAs, especially short- and medium-chain fatty acids (SCFAs and MCFAs), are the major FAs in the kernel oils. SCFAs and MCFAs present a backbone between 4 and 16 FAs. They are mostly found in lauric fats such as coconut, babassu, and palm kernel. In addition to their crucial role as an energy source, SCFAs and MCFAs also show faster oxidation in the liver, and they have been associated with glucose modulation, hormone secretion, lipid metabolism, and heat production. Diets containing those FAs have been widely studied for their potential benefits to human health and their association with metabolic disorders [189].

**Figure 7:**
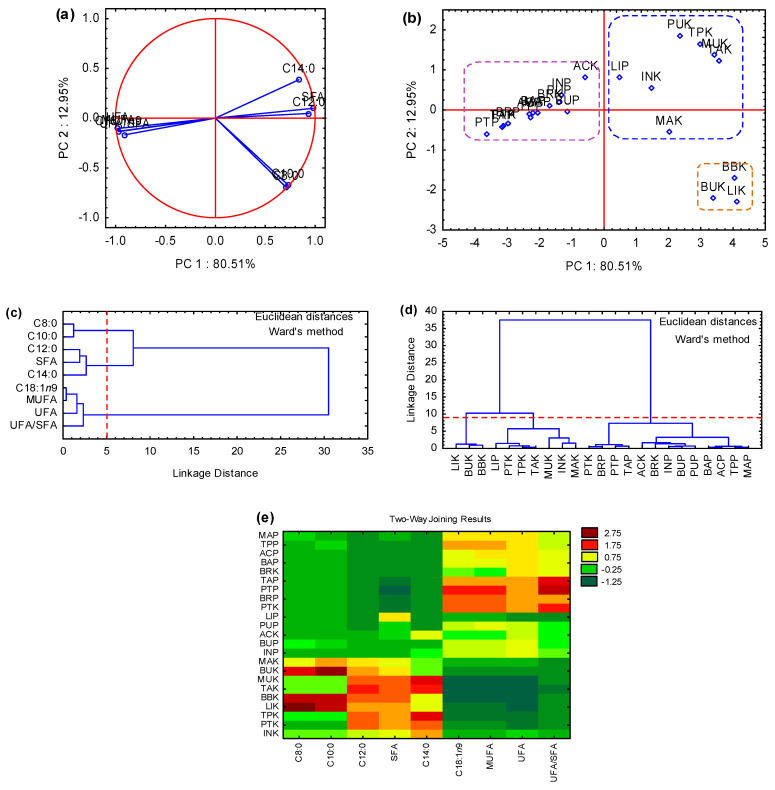
Principal component analysis (PCA) and hierarchical cluster analysis (HCA) of Arecaceae palm fruit oils based on their fatty acid profile. Loading plot for principal component 1 × principal component 2 with the projection of variables (**a**) and score plot of oil samples (**b**); clustering and linkage distance of variables (**c**) and oil samples (**d**) from HCA based on Ward’s method; and the heatmap showing the similarities and differences among oils according to the main significant variables (**e**). Refer to Table 1 for samples’ acronyms.

Multivariate statistical analysis was assessed to identify similarities among the APTFOs using the mean values of their fatty acids and oil content, totaling 30 variables. Principal component analysis (PCA), hierarchical clustering analysis (HCA), and two-way joining analysis were used to verify possible relationships among 23 oils from both pulp and kernel (Figure 7).

The preliminary evaluation showed that 21 variables did not contribute significantly to establishing correlations among the studied oils. Thus, the assessment was based on nine variables whose contribution was higher than 70% to explain the data variability: five fatty acids (C8:0, C10:0, C12:0, C14:0, and C18:1n9), SFA, MUFA, UFA, and the ratio between UFA and SFA.

Figure 7a depicts the projection of the significant variables in a 2D plot where it is possible to observe that PC1 and PC2 were responsible for explaining 80.15 and 12.95% of the data variability. Figure 7b shows that the APTFOs can be grouped based on the similarities between their FAs. HCA confirmed the results from PCA and indicated that both variables and the oils were mainly grouped in three clusters. The correlation among the composition of the oils suggested that FAs such as capric, caprylic, lauric, and myristic acids are the SFAs in APTFOs. On the other hand, oleic acid (C18:1*n*9) was the major contributor to grouping connections related to unsaturated FAs among the oils (Figure 7c).

HCA also showed a high correlation among the properties of kernel oils versus pulp oils, which were also grouped in three clusters (Figure 7d). The heatmap confirms the findings from PCA and HCA, showing the main correlations among APTFOs and the main variables responsible for their similarities (Figure 7e). The kernel oils from BBK, BUK, and LIK were grouped in the positive quadrant of PC1, indicating high correlations among their FA profiles. LIP showed similar properties to APTFOs from the kernels. On the other hand, the oils PTK, ACK, and BRK also displayed a correlation with those obtained from pulps, indicating a non-standard behavior regarding the fatty acid profile of Arecaceae palm fruit oils.

## 7. Biological Effects Reported for APTFOs

The biological effects and potential applications for APTFOs have been reported in the literature. Nunes et al. [190] studied the impact of feeding diabetic rats with MKO as a partial substitute for carbohydrates. The authors reported a decrease in hyperglycemia, an improvement in insulin secretion, and a reduction in insulin resistance.

Santos et al. [191] reported that the oils from *A. aculeatum*, *O. bataua*, *M. flexuosa*, and *A. speciosa* have insecticidal activity against maize weevil (*Sitophilus zeamais* Motschulsky), suggesting its use as an alternative to harmful pesticides. Crude and hydrolyzed MUK butter have been evaluated for their antimicrobial activity. The oils have shown action against *Clostridium perfringens*, *Enterococcus cecorum*, *Listeria monocytogenes*, and *Staphylococcus aureus* at 0.14–4.5 mg mL^−1^ [85].

The wound healing potential of a topical herbal formulation with BRP oil and MUK butter was evaluated. The accelerated wound healing in a rat skin model was observed, and the prediction of skin sensitization in silico showed that none of the identified compounds had any potential skin toxicity for humans [192]. Cosmetic nanoemulsions containing ACP oil were produced and considered suitable as a vehicle for imidazole antifungals [150]. Promising results were also found for nanoemulsions containing ACP oil as a new source of photosensitizing molecules to treat melanomas [193]. Although large progress in the industrial use of ACP oil has been verified, the knowledge of the potential applications of ACK oil is scarce. A study suggested that ACK oil shows tyrosinase inhibitory activity and may be used to treat hyperpigmentation and other related disorders [159].

The antioxidant potential and modulatory effects of MUK butter on human hepatoma cells (HepG2) before and after lipase interesterification were reported. Results indicate that the enzymatic interesterification did not change the concentration of bioactive compounds and the antioxidant activity of MUK butter. In addition, the restructured lipids modulated the HepG2 endogenous antioxidant enzyme [194].

Nanocapsules loaded with TPP oil showed antioxidant activity and antiproliferative potential against MCF-7 (human breast cancer cell line). Such anticancer activity may be related to the antioxidant activity of TPP oil, which is due to the presence of β-sitosterol, squalene (phytosterols), all-*trans*-β-carotene, and oleic acid [149]. *A. vulgare* oil nanocapsules were used to feed silver catfish *Rhamdia quelen,* and the results indicated that the oil could be an alternative non-toxic lipid source for fishes [195].

The use of TPP oil in alloxan-induced diabetic mice to treat hyperglycemia was reported, and the results indicated its antihyperglycemic and antioxidant activities [160]. Another study also showed that TPP oil might prevent changes in serum enzymes of the purinergic system, resulting in an improvement of the immune system [196]. Furthermore, TPP oil showed its effectiveness in the hepatoprotection against lipid oxidative damage and in the prevention of lipid peroxidation in hepatic tissue [197]. Antihyperglycemic and antioxidant activities in alloxan-induced diabetic mice were also reported [160].

ACP oil has been used to feed Nile tilapia (*Oreochromis niloticus*), and the results indicated that the diet containing this oil stimulated antioxidant responses. A reduction in lipid peroxidation and free radical formation in the liver of the animals were observed. Nevertheless, negative impacts on the hepatic metabolism of the fish were observed [198].

The antibacterial properties of BRP oil were investigated [199], and enzymatic hydrolysis has been used to produce ω-6 and ω-9 from BRP oil [200]. The antioxidant capacity of BRP oil has been shown [172]. The oleic acid content in BRP oil has been associated with a modulator of aminoglycoside activity, indicating antibiotic activity against methicillin-resistant *S. aureus* [201]. Meat quality and FA composition of lambs fed with BBK and BRP oils were evaluated, resulting in reduced weight of fat depots in the animals [202]. BRP oil has been assessed as an efficient insecticidal against maize weevil (*S. zeamais*) [191].

Current research on the application of LIK oil includes its use as an adjuvant to treat inflammations caused by *Propionibacterium acne* [203]. Souza et al. [163] reported in a study with mice that LIK oil had no significant toxic effects and did not show genetic damage, indicating that it is safe for human consumption. LIK oil consumption was evaluated in animal models, and doses below 2 g kg^−1^ were considered safe. LIK oil also significantly reduced the side effects caused by indomethacin, a powerful anti-inflammatory, preventing necrosis and decreasing inflammatory processes [128]. The addition of LIK oil in the diet of goats up to 4.5% did not cause significant changes in the carcass [204] and the milk’s physicochemical properties [205]. Another study found that up to 4.5% LIK oil in the diet of prepubertal male goats did not alter testicular development based on testicular biometry and morphometry [206]. These findings indicated that LIK oil could be an alternative lipid source for animal nutrition in the semi-arid regions of northeastern Brazil [204,205]. The use of 4.5% LIK as a supplement for improving the quality of cow’s milk was also evaluated. The authors indicated that it could be a viable lipid source to enhance the nutritional quality of the milk [207,208].

## 8. Potential Applications of APTFOs

The reported applications of APTFOs are displayed in Table 6. The effect of using MUK butter as nanostructured lipid carriers for β-carotene and α-tocopherol was evaluated and indicated its suitability for the controlled release of bioactive compounds [209]. Former industrial applications of MUK butter included the production of soaps and margarine in São Paulo, the United States, and Europe in the 1950s [38,132]. MUK butter was also used as feedstock for biodiesel [210]. However, current uses of MUK butter in Brazil are restricted to cosmetics such as skin care creams, shampoos, moisturizers, soaps, conditioners, moisturizing facial and hair masks, combing cream, hair butter, and leave-in, solid shampoo, serum, hair cleaning cream, and hair balm. The emollient capacity of MUK butter has been confirmed in cosmetic model emulsions [211]. Speranza, Ribeiro, and Macedo [86] reported that enzymatic interesterification might favor the development of lipid fractions from MUK butter with enhanced properties for cosmetics and pharmaceutical applications.

In cosmetics, TAK oil has been employed as the lipid phase in nanocapsules [149,195] and biodiesel production via the alcohol route [212]. TPK oil was also successfully used to produce biodiesel using methanol and ethanol [44]. Biodiesel production is also among the industrial applications suggested for BRP oil [19,213]. Many authors reported LIK oil as a raw material for biofuel production [18,68,153,154,214]. Araújo et al. [154] indicated that LIK oil has excellent properties for jet fuel, as 90% of its composition fits well with the requirements for this product.

The application of BBK oil includes model emulsions [215], cosmetics and pharmaceutical nanoemulsions [216], skin moisturizers [217], biodiesel, and cosmetics [218]. The biodiesel production from BBK oil using ethanol and methanol has been extensively investigated. The authors suggest a high potential as an alternative for regular production of biodiesel due to its availability [168]. In addition to the reported uses of BBK, it is also largely used as a frying oil in Maranhão and Pará States in Brazil. Faria et al. [52] suggested using BUK oil as an alternative for the formulation of foodstuffs and animal feed due to its high content of medium-chain SFAs and oleic acid.

The use of ACP oil as an active ingredient with enhanced properties and high added value in many foodstuffs, pharmaceuticals, biofuels, animal feed, and cosmetics has been reported [182,198,219]. ACP oil was used as the lipid phase in multiple emulsions, which was developed as an alternative lotion with a sun protection factor [220]. Poly (urethane-urea) nanoparticles based on ACP oil produced by miniemulsion polymerization were developed, and the results suggested the use of such nanoparticles for the controlled and targeted delivery of therapeutics [219].

**Table 6:** Potential applications of Arecaceae palm tree fruit oils.

Oil	Potential Application	Reference
MUK butter	Nanostructured lipid carriers for β-carotene and α-tocopherol	[209]
	Soaps and margarine	[38,132]
	Biodiesel	[210]
	Cosmetic model emulsions	[211]
	Interesterified lipid fractions	[86]
	Skin care creams, shampoos, moisturizers, soaps, conditioners, moisturizing facial and hair masks, combing cream, hair butter, and leave-in, solid shampoo, serum, hair cleaning cream, and hair balm	This study
TAK oil	Cosmetic nanocapsules	[149,195]
	Biodiesel	[212]
TPK oil	Biodiesel	[44]
BRP oil	Biodiesel	[19,213]
LIK oil	Biofuel	[18,68,153,154,214]
	Moisturizer	[221]
	Biofilms for food packaging	[222]
	Bio-oil	[214]
BBK oil	Cosmetic model emulsion for skin	[215]
	Cosmetics and pharmaceutical nanoemulsions	[216]
	Skin moisturizers	[217]
	Biodiesel and cosmetics	[168,218]
	Frying oil	This study
BUK oil	Foodstuffs and animal feed	[52]
ACP oil	Extracts with cytotoxicity on HT-29 colon cancer cells	[182]
	Feed for Nile tilapia (*Oreochromis niloticus*)	[198]
	Polyurethane nanoparticles for controlled and targeted delivery of therapeutics	[219]
	Lotion with sun protection factor	[220]

MUK, murumuru kernel (*A. murumuru*); TAK, tucumã-do-Amazonas kernel (*A. aculeatum*); BPR, buriti pulp (*M. flexuosa*); LIK, licuri kernel (*S. coronata*); BBK, babassu kernel (*A. speciosa*); BUK, butiá kernel (*Butia* spp.); ACP, açaí pulp (*E. oleracea*).

Cosmetic emulsions based on 10% LIK oil as the lipid phase showed significant hydration power. The authors suggest that LIK oil can be used as an alternative to sweet almond oil in common moisturizer formulations [221]. The addition of LIK oil to biofilms based on arrowroot starch caused significant and positive effects on its mechanical, solubility, and color properties, indicating that it may be an alternative and sustainable raw material for the development of biofilms used in biodegradable packaging [222]. Oliveira et al. [214] studied the properties and applicability of vermiculite clay as an alternative catalyst and catalytic support for zinc oxide in the pyrolysis of LIK oil, suggesting the formation of a bio-oil rich in hydrocarbons.

## 9. Co-Products from the Oil Extraction of Arecaceae Palm Fruits

The main co-products resulting from the oil extraction of Arecaceae palm fruits are highlighted in Table 7. The extraction of oils from APTFs results in high volumes of waste and coproducts such as press-cakes, shells, skin, and defatted flour. Although many studies have been reported in the literature on the utilization of co-products from APTFOs extraction, there is not usually a proper destination for their residues and/or conversion of the waste into coproducts.

Toledo e Silva et al. [223] reported the amino acid profile of the cake from MAK. Results indicated that it is rich in globulins 11 S and 7 S (58.5% of storage proteins) composed of essential amino acids such as methionine and cysteine. On the other hand, the endocarp from macaúba has been employed for producing activated carbon using vacuum pyrolysis. Furthermore, the alternative biochar was successfully used to remove atrazine, an herbicide widely used and considered an emerging pollutant in surface water and groundwater [224].

The seeds of tucumã-do-Amazonas, usually discarded without adequate treatment, were used as a raw material to produce activated [225] and nanomagnetic activated carbon [226]. The peels of tucumã-do-Amazonas were also successfully employed as catalysts for biodiesel production, providing about 97% efficiency on biodiesel conversion with only 1% (*w/w*) catalyst [227]. The hydro-alcoholic extracts obtained from both the pulp and peel of tucumã-do-Amazonas showed antibacterial activity against *Enterococcus faecalis*, *Bacillus cereus*, and *Listeria monocytogenes* and antifungal activity against *Candida albicans* [228]. Ethanolic extracts from the pulp and peel of tucumã-do-Amazonas have shown positive effects on preventing memory loss and oxidative damage in rats with hyperlipidemia [229] and anti-inflammatory activity and antioxidant properties in cell cultures [82]. It was also verified that the ethanolic extract has no cytotoxic effect in Wistar rats [230], suggesting its safety for consumption or use as an ingredient in food formulations.

The cake from the extraction of MUK butter has been used as an alternative to the dietary supplementation of ruminants in replacing Mombasa grass [231]. MUK shell has been used to synthesize sulfonated catalysts for biodiesel production [21]. Silva et al. [232] showed that the coproducts from inajá (*A. maripa*) processing are rich in phenolic compounds, mainly flavan-3-ols. Extracts from inajá cake rich in catechins and procyanidins showed significant peroxyl radical scavenging capacity. Moreover, they inhibit the activation of NF-κB activation, an essential transcription factor for inflammatory responses and one of the most important molecules linking chronic inflammation to cancer [233].

**Table 7:** Co-products from the oil extraction of Arecaceae palm fruits.

Raw Material	Co-Product	Reference
Macaúba kernel	Press-cake rich in essential amino acids	[223]
	Activated carbon	[224]
Tucumã-do-Amazonas seeds	Activated and nanomagnetic activated carbon	[225,226]
Tucumã-do-Amazonas peel	Biodiesel	[227]
Tucumã-do-Amazonas peel and pulp	Extracts with antifungal and antibacterial activity	[228]
	Extracts with antioxidant and anti-inflammatory activity	[82,229]
	Extracts with no cytotoxic effects in Wistar rats	[230]
Babassu mesocarp	Mesocarp powder	[234]
	Enzymes (lipase, protease, amylase) production	[235,236]
	Ethanol	[237]
*Butia* spp. kernels	Ingredient for foodstuffs (sweets, bread, cakes, pies, and cookies)	[52]
Açaí seeds	Edible flour with potential anti-obesity properties	[143]
	Extracts with antimicrobial and antioxidant properties	[238]
	Dietary fiber and antioxidant-rich extracts	[56]

The mesocarp of babassu and the cake obtained from BBK oil extraction are popularly used to treat gastritis, leukorrhea, and wounds [234]. The press-cake has also been used as a substrate to produce enzymes such as lipases, proteases, and amylases from *Penicillium restrictum* fungus [235,236]. The hydrolyzed and fermented mesocarp of babassu has been used to obtain ethanol [237].

Butiá (*B. capitata* var *capitata*) kernel, rich in fibers and minerals such as phosphorus, potassium, magnesium, and sulfur, has been used as an ingredient for sweets, bread, cakes, pies, and cookies. The results showed that it could enhance the texture, flavor, and nutritional properties of these foodstuffs [52]. The processing of açaí generates tons of residues, mostly seeds. Most studies involving the use of açaí seeds were focused on obtaining flour and antioxidant-rich extracts for nutritional enrichment and therapeutic uses [143,238].

The addition of 17% ground licuri in the diet of lambs improved feed conversion and increased average daily gain [239]. However, a decrease in lamb performance due to decreased dry matter intake was observed [240]. The lambs fed with 16% licuri cake had no changes in the dry matter intake [241]. Nonetheless, the addition of 24% licuri cake in the feed of lambs resulted in a 39% reduction in intake, decrease in weight gain, and average daily gain [242]. On the other hand, the addition of 45% of licuri cake did not affect the intake or digestibility of Boer goats [243]. Licuri cake, which is rich in linoleic, oleic, and palmitic acids (35.3, 21.5, and 17.6%, respectively), improved the FA profile and the sensory attributes of lamb meat [244]. Furthermore, the licuri nutshell has been reported as a potential source of biomass for energy production based on fuel [245].

## 10. Concluding Remarks

The high occurrence of Arecaceae palm trees in South America, especially in Brazilian biomes, indicates a great opportunity for their sustainable exploitation. The recovery of lipids and other coproducts from their fruits has been a growing trend in the last two decades, indicating an increasing interest in valorizing such raw materials. Most of the APTFOs discussed in this review are not commercially available or do not have their main characteristics fully assessed. Multivariate analysis suggested a correlation between the composition of pulp and kernel oils. The main SFAs and UFAs observed in APTFOs were myristic and oleic acid, respectively. Due to high international trading volumes and current industrial applications, babassu oil (BBK) has been considered the most economically relevant. Açaí pulp oil (ACP), murumuru kernel butter (MUK), and patawa pulp oil (PTP) are currently sold worldwide due to partnerships between extractivists which are represented by cooperatives and e-commerce platforms. For instance, some initiatives from small producers like the Amazon Oil company currently valorize the APTFs sustainably collected by partners and extractivists to obtain oils and fats. The increase of partnerships between the local population and companies and/or cooperatives can expand the local economy, improving the social conditions of people who survive from extractivism from these species while contributing to the preservation of natural resources. APTFOs are rich in bioactive compounds such as carotenoids and phenolic compounds, and they present desirable characteristics for formulating cosmetics, pharmaceuticals, and biofuels. In order to use APTFOs for food purposes safely, prior cytotoxic evaluations are required. More studies on the physicochemical properties, TAG composition, stability, bioactive compounds, biological properties, antioxidant activity, cytotoxicity, and application of some APTFOs are also needed. These findings may help understand the main features of APTFOs and may contribute to their spreading. These native palm trees, which are a valuable source of specialty oils and coproducts, can be valorized, and in this way, the risk of extinction for some of them may no longer be a problem in Brazil.

## Figures and Tables

**Table 1:** Nomenclature and oil content of emerging Arecaceae palms.

Scientific Name	Common Name	Oil Content (%)		Raw Material Code	References
*Acrocomia aculeata*	Macaúba	Pulp	9.77–28.94	MAP	[28,29]
		Kernel	3.43–47.50	MAK	[29,30,31]
*Astrocaryum aculeatum*	Tucumã-do-Amazonas	Pulp	21.25–61.60	TAP	[32,33,34]
		Kernel	28.40–45.50	TAK	[35]
*Astrocaryum murumuru*	Murumuru	Pulp	2.60	MUP	[36]
		Kernel	27.70–44.00	MUK	[37,38]
*Astrocaryum vulgare*	Tucumã-do-Pará	Pulp	1.90–40.49	TPP	[12,37,39,40]
		Kernel	9.60–29.59	TPK	[37,39]
*Attalea maripa*	Inajá	Pulp	35.52–56.20	INP	[12,41]
		Kernel	31.30–67.69	INK	[39,42,43]
*Attalea speciosa*	Babassu	Kernel	50.31–66.00	BBK	[44,45]
*Bactris gasipaes*	Pupunha	Pulp	7.70–61.70	PUP	[46,47,48]
		Kernel	11.50–23.50	PUK	[39,48,49]
*Butia* spp.	Butiá	Pulp	2.60–2.73	BUP	[50,51]
		Kernel	31.96–53.60	BUK	[52,53]
*Euterpe oleracea*	Açaí	Pulp	7.00–48.00	ACP	[54]
		Kernel	0.22–2.84	ACK	[55,56]
*Mauritia flexuosa*	Buriti	Pulp	19.00–51.67	BRP	[12,57,58]
		Kernel	7.01–9.20	BRK	[59]
*Oenocarpus bataua*	Patauá	Pulp	14.40–51.60	PTP	[12,57,60]
		Kernel	0.06–1.30	PTK	[37,61]
*Oenocarpus bacaba*	Bacaba	Pulp	7.40–60.39	BAP	[62,63,64]
		Kernel	0.83–4.10	BAK	[37,61]
*Syagrus coronata*	Licuri	Pulp	4.11–4.50	LIP	[65,66,67]
		Kernel	39.00–50.00	LIK	[65,66,68,69]

**Table 2:** Physicochemical properties of pulp and kernel oils from Arecaceae palm fruits.

Oil	FFA (% Oleic Acid)	AV (mg KOH g^−1^)	PV (mEq O_2_ kg^−1^)	IV (g I_2_ 100 g^−1^)	SV (mg KOH g^−1^)	RI, 40 °C	UM (%)	OSI, 110 °C (h)	MP (°C)	KV, 40 °C (mm^2^ s^−1^)	ρ, 40 °C (kg m^−3^)	References
MAP	1.72–5.63	-	3.16–5.0	-	-	-	-	16	-	-	-	[31,72,146]
MAK	0.94–4.9	112	1.9–6.9	25.5–30.9	230.0–235.4	-	-	0.07–26	-	41.9	919 ^e^	[30,68]
TAP	-	19.10–20.63	11.8	10.88	196.1–298.39	-	-	-	19.1–20.3	41.8–42.2	-	[32,134]
TAK	0.1–0.17 ^a^	0.26–0.48	-	08-11	208.60–246.00	-	-	20–44	19.5–33.0	44.2–46.1	-	[35,134]
MUK	0.36	0.8 ^b^–5.16	5.0 ^b^–22.85	11.0 ^b^–13.12	228.3 ^b^–258.24	1.45 ^b^–1.46	0.11 ^b^–0.16	18 ^d^–40.0 ^b^	25–37	29	904.17–907.8 ^e^	[10,11,19,147]
TPP	0.7–2.75	3.80–15.25	0.5–10.52	67.77–73.6	180.40–202.71	1.43–1.47	1.8–2.2	10.1 ^c^	13.8	-	910 ^c^–975	[37,40,135,148,149]
TPK	0.1	0.45–10.91	1.19–9.0	12.05–18.07	236.40–246.43	1.43–1.45	0.47–1.1	3.87 ^d^	30.3	15.40–30.5	860–904.15	[10,37,39,135]
INP	1	-	6.5–176	74.15–74.86	210–214	-	0.8	6.3 ^c^	-	-		[42,148]
INK	4.85–5.06	2.6	4.0–25	16.48–16.79	262–263	1.45	0.6	-	-	-	930	[39,42]
BBK	0.05–0.08 ^a^	1.06	0.22–0.91	10.36	248.73–249.50	1.45–1.47	-	2–17 ^d^	23.9–26.52	-	918.63–919.10 ^e^	[11,94,138]
PUP	2	2.45	5.47–6.4	-	-	-	1.3	34.2 ^c^	-	-	-	[47,148]
PUK	-	1.70–12.20	68.6	-	-	1.45	0.8–3.0	-	-	-	900	[39,49]
ACP	-	3.66 ^b^	-	71 ^b^	199 ^b^	1.46 ^b^	-	-	-	-	952 ^e^	[150]
ACK	-	93.1	38.46–62.05	91.3	186.1	1.45	-	-	-	-	924.3 ^e^	[55]
BRP	1.14–6.22	2.13–5.76	7.4–14.20	70.17–77.40	183.91–192.88	1.46–1.47	0.5–1.3	16.9 ^c^-18.3	37.2 ^b^-25.1	40.8	921.2 ^e^	[11,19,59,148,151]
BRK	-	-	-	-	-	-	-	3.55–6.91	-	-	-	[59]
PTP	0.4	-	18.3	76.3	-	1.46	0.8	-	<8.0	-	-	[37]
PTK	-	14.52	15.94	75.85	174.12	-	0.81	4.97 ^d^	-	38.96	900.03	[10,37]
BAP	2.4–63.0	1.65	11.9–76.1	51.35–75.8	225	1.46	1.0–2.6	5.65–11.9 ^c^	32.6	-	-	[37,64,148,152]
BAK	35.6	-	-	80.9	-	1.45	-	-	<10.0	-	-	[37]
LIK	1.4	0.72–1.5	-	13.9–18.5	-	-	-	10.7–69.6	-	23.4–27.6	920 ^e^–924 ^e^	[68,153,154]

FFA, free fatty acids; AV, acid value; PV, peroxide value; IV, iodine value; SV, saponification value; RI, refractive index; UM, unsaponifiable matter; OSI, oxidative stability index; MP, melting point; KV, kinematic viscosity; ρ, density. ^a^ % lauric acid. ^b^ Refined oil. ^c^ OSI at 100 °C; ^d^ OSI at 130 °C. ^e^ Density at 20 °C. Oils’ acronyms: MAP and MAK, macaúba pulp and kernel oils (*A. aculeata*); TAP and TAK, tucumã-do-Amazonas pulp and kernel oils (*A. aculeatum*); MUK, murumuru kernel butter (*A. murumuru*); TPP and TPK, tucumã-do-Pará pulp and kernel oils (*A. vulgare*); INP and INK, inajá pulp and kernel oils (*A. maripa*); BBK, babassu kernel oil (*A. speciosa*); PUP and PUK, peach palm pulp and kernel oils (*B. gasipaes*); ACP and ACK, açaí pulp and kernel oils (*E. oleracea*); BRP and BRK, buriti pulp and kernel oils (*M. flexuosa*); PTP and PTK, patawa pulp and kernel oils (*O. bataua*); BAP and BAK, bacaba pulp and kernel oils (*O. bacaba*); LIK, licuri kernel oil (*S. coronata*).

**Table 3:** Fatty acid (FA) composition (%) of different oils from pulp and kernels of Arecaceae palm fruits.

Pulp Oils												
FAs		MAP	TAP	TPP	INP	PUP	BUP	ACP	BRP	PTP	BAP	LIP
C6:0		0.1	-	-	-	-	6.73–6.88	-	-	-	-	-
C7:0		0.1	-	-	-	-	-	-	-	-	-	-
C8:0		0.1–0.3	-	-	-	-	1.28–1.42	-	-	-	-	-
C9:0		0.23	-	-	-	-	-	-	-	-	-	-
C10:0		0.07–0.10	-	0.80	-	-	0.29–0.40	-	-	-	-	-
C11:0		0.04	-	-	-	-	-	-	-	-	-	-
C12:0		0.08–0.26	0.20	-	0.68–4.60	-	2.71–3.64	0.04–0.08	0.02–0.10	-	-	-
C13:0		0.03	-	-	-	-	-	-	-	-	-	-
C14:0		0.02–0.64	0.50	0.10	1.57–10.70	0.10	3.05–3.55	0.11–0.14	0.10–0.12	-	0.15–0.29	-
C15:0		0.08	-	-	-	-	-	-	0.04	-	-	-
C16:0		23.37–29.75	7.89–10.40	21.20–24.60	19.84–25.10	24.10–50.57	18.81–19.35	22.80–26.78	17.59–20.80	11.3–12.2	17.19–25.90	42.31
C16:1n7		5.21	-	0.10	0.10–0.30	3.39–7.40	0.48–0.49	4.20–5.40	0.25–0.26	0.6–0.7	0.68–1.10	-
C17:0		0.29	1.70	0.10	-	0.11	-	-	0.05–0.08	-	-	-
C17:1		-	-	-	-	-	-	-	-	-	-	-
C18:0		1.31–4.23	4.90–6.72	2.60–5.10	1.60–3.50	0.80–3.50	1.82–2.00	1.40–1.90	1.35–1.60	3.1–3.9	2.19–4.70	32.35
C18:1n9		54.79–63.21	64.20–73.81	64.7–67.62	39.20–60.82	36.27–60.80	47.33–48.07	51.46–62.30	71.60–78.55	77.7–79.1	46.20–66.24	25.34
C18:1n11		-	-	-	-	-	-	3.39–4.80	-		-	-
C18:2*n*6		5.42–10.26	11.00–12.14	1.15–5.30	6.47–12.90	2.50–5.40	11.00–12.17	7.28–10.60	1.13–2.50	3.6–4.9	12.12–20.00	-
C18:3*n*3		1.32–3.19	1.00	0.20–3.10	0.20–6.60	1.20–1.40	3.13–3.66	0.30–0.81	0.09–1.40	0.5–0.6	0.58–0.72	-
C20:0		0.17	0.40–1.89	4.10	3.20	-	0.16–0.21	0.10–0.12	0.66–1.65	0.1	0.06–0.19	-
C20:1*n*9		-	-	-	1.30	-	0.10–0.13	-	0.70	-	-	-
C22:0		-	0.10	0.10	-	-	0.07–0.09	-	-	-	0.01–0.07	-
Σ SFA		24.97–36.29	13.19–21.51	23.80–34.90	23.69–47.10	24.90–50.57	34.92–37.54	26.70–28.84	19.77–24.39	14.40–16.20	19.60–31.15	74.66
Σ MUFA		54.79–68.42	64.20–73.81	64.70–67.62	39.30–62.42	46.70–68.20	47.91–48.69	59.73–62.30	71.85–79.51	78.30–79.80	44.88–67.34	25.34
Σ PUFA		6.74–13.45	11.00–13.14	1.35–8.40	6.67–19.50	3.70–6.80	14.13–15.83	7.83–11.10	1.22–3.90	4.10–5.50	12.70–20.72	0.00
References		[28,31]	[34,134]	[12,148,159,160]	[12,41,136,148]	[46,47,148]	[51]	[139,141,159]	[11,12,148]	[10,11,12,57,159]	[63,148]	[161]
**Kernel oils**												
**FAs**	**MAK**	**TAK**	**MUK**	**TPK**	**INK**	**BBK**	**PUK**	**BUK**	**ACK**	**BRK**	**PTK**	**LIK**
C6:0	0.03–0.80	-	-	-	-	0.54–0.55	-	0.4	-	-	-	-
C7:0	0.01	-	-	-	-	-	-	-	-	-	-	-
C8:0	1.06–6.20	1.80–2.20	1.19–2.70	1.94	3.80	7.50–8.57	-	7.8	-	-	0.02	9.00–13.00
C9:0	0.32	-	-	-	-	-	-	-	-	-	-	-
C10:0	3.94–4.20	1.80–2.30	1.31–2.00	1.95	4.00	6.55–7.42	-	8.0	-	-	0.03	5.80–8.00
C11:0	0.12	-	-	-	-	2.10–2.22	-	-	-	-	-	-
C12:0	31.96–45.70	52.30–57.50	47.15–51.60	43.50–50.89	10.06–40.50	47.62–49.97	33.30–60.60	42.1	2.90–8.74	-	0.20–1.37	36.10–48.00
C13:0	0.14	-	0.08	-	-	0.03–1.51	-	-	-	-	0.10–0.19	-
C14:0	8.30–12.14	23.40–27.10	25.80–28.75	25.20–28.60	13.59–25.50	13.85–14.22	18.90–27.80	10.5	4.60–22.86	-	0.09–0.94	14.00–16.40
C15:0	0.47	-	-	-	-	-	-	-	-	-	0.30–0.50	-
C16:0	6.90–11.11	4.70–5.90	6.00–7.09	6.23–7.50	9.00–20.55	6.59–7.30	6.00–9.60	6.0	16.10–16.27	18.07–24.28	9.68–25.96	5.00–8.80
C16:1*n*7	0.29	-	-	-	0.16–0.29	0.21–0.23	-	-	0.61	-	0.31–1.61	-
C17:0	0.35	-	-	-	-	0.01–0.06	-	-	-	-	0.06–0.10	-
C17:1	-	-	-	-	-	-	-	-	-	-	0.05–0.11	
C18:0	2.40–6.63	1.90–2.70	2.57–2.92	2.74	1.85–2.40	2.69–3.31	5.10	4.0	1.37–3.20	3.83–5.50	0.87–5.09	2.00–4.00
C18:1*n*9	24.10–29.10	5.0–7.0	5.70–7.97	8.10–13.60	10.80–43.39	8.60–9.57	12.90–24.30	16.9	26.79–51.30	27.59–52.96	64.78–81.91	7.00–12.00
C18:1*n*11	-	-	-	-	-	-	-	-	-	-	0.97–3.41	-
C18:2*n*6	2.85–3.40	2.1–3.1	3.00–3.62	2.94–3.30	2.40–6.96	0.27–1.52	-	4.2	14.60–25.52	16.24–36.04	1.18–5.97	3.00–3.10
C18:3*n*3	-	-	0.12	-	7.62–7.81	1.36–1.64	-	-	0.84	1.74–13.33	0.08–1.21	-
C20:0	0.33	0.08–0.11	0.10	-	-	-	-	0.1	2.30	-	0.03–0.60	-
C20:1*n*9	-	0.05	-	-	-		-	0.04	1.50	-	0.07–0.13	-
C21:0	-	-	-	-	-	0.04	-	-	-	-	-	-
C22:0	0.12	0.06	0.03	-	-	-	-	-	-	-	-	-
C24:0	0.19	0.04–0.07	-	-	-	-	-	-	-	-	-	-
Σ SFA	54.59–88.83	85.98–97.87	84.02–95.27	74.93–93.62	34.50–96.75	84.48–95.17	75.70–85.50	78.90	29.10	21.90–29.78	11.33–34.80	71.90–98.20
Σ MUFA	24.10–29.39	5.00–7.05	6.85–7.97	8.10–13.60	10.96–43.68	8.81–9.79	12.90	16.94	52.80	27.59–52.96	66.18–87.17	7.00–12.00
Σ PUFA	2.85–3.40	2.10–3.10	3.26–3.62	2.94–3.30	10.02–14-77	1.52–1.95	0.00	4.20	14.60	17.98–49.37	1.26–7.18	3.00–3.10
References	[30,31,162]	[35]	[10,11,37]	[10,39]	[39,42]	[11,138]	[39,49]	[52]	[55,56]	[59]	[10,11,12,57,159]	[68,153,154,163]

Fatty acids: C6:0, caproic; C7:0, heptanoic; C8:0, caprylic; C9:0, nonanoic; C10:0, capric; C11:0, undecanoic; C12:0, lauric; C13:0, tridecanoic; C14:0, myristic; C15:0, pentadecanoic; C16:0, palmitic; C16:1*n*7, palmitoleic; C17:0, heptadecanoic; C17:1, heptadecenoic; C18:0, stearic; C18:1*n*9, oleic; C18:1*n*11, vaccenic; C18:2*n*6, linoleic; C18:3*n*3, linolenic; C20:0, arachidic; C20:1*n*9, gondoic; C21:0, heneicosanoic; C22:0, behenic; C24:0, lignoceric. SFA, saturated fatty acids; MUFA, monounsaturated fatty acids. PUFA, polyunsaturated fatty acids. Samples’ acronyms: MAP and MAK, macaúba pulp and kernel oils (*A. aculeata*); TAP and TAK, tucumã-do-Amazonas pulp and kernel oils (*A. aculeatum*); MUK, murumuru kernel butter (*A. murumuru*); TPP and TPK, tucumã-do-Pará pulp and kernel oils (*A. vulgare*); INP and INK, inajá pulp and kernel oils (*A. maripa*); BBK, babassu kernel oil (*A. speciosa*); PUP and PUK, peach palm pulp and kernel oils (*B. gasipaes*); BUP and BUK, butiá pulp and kernel oils (*Butia* spp.); ACP and ACK, açaí pulp and kernel oils (*E. oleracea*); BRP and BRK, buriti pulp and kernel oils (*M. flexuosa*); PTP and PTK, patawa pulp and kernel oils (*O. bataua*); BAP, bacaba pulp oil (*O. bacaba*); LIP and LIK, licuri pulp and kernel oils (*S. coronata*).

**Table 4:** Triacylglycerol (TAG) composition* of different Arecaceae palm fruit oils.

TAG	MUK	TPP	TPK	INP	BBK	PUP	BRP	PTP	BAP	LIK
CCC	-	-	-	-	-	-	-	-	-	3.12
CLM	-	-	-	-	-	-	-	-	-	14.47
CLL	-	-	-	-	-	-	-	-	-	21.03
COL	-	-	-	-	4.54–4.95	-	-	-	-	-
CpCpC	-	-	-	-	0.41–0.44	-	-	-	-	-
CpCpL	-	-	-	-	1.57–1.69	-	-	-	-	-
CpLC	-	-	-	-	2.80–3.01	-	-	-	-	-
CpLM	-	-	-	-	-	-	-	-	-	4.54
CpLL	-	-	-	-	-	-	-	-	-	25.76
CpLP	-	-	-	-	-	-	-	-	-	4.55
CpLO	-	-	-	-	-	-	-	-	-	2.76
CpOL	-	-	-	-	4.48–4.91	-	-	-	-	-
CpOC	-	-	-	-	1.10–1.19	-	-	-	-	-
CpOCp	-	-	-	-	0.49–0.53	-	-	-	-	-
CpLnL	-	-	-	-	0.82	-	-	-	-	-
LLL	13.73	-	19.52	-	14.46–15.13	-	-	-	-	-
LLM	14.69	-	22.87	-	10.33–10.72	-	-	-	-	11.53
LLC	-	-	-	-	8.45–8.95	-	-	-	-	-
LLCp	-	-	-	-	7.67–8.11	-	-	-	-	-
LLO	-	-	5.91	-	-	-	-	-	-	1.86
LLP	7.15	-	-	-	6.54–7.04	-	-	-	-	-
LLS	-	-	-	-	3.63–4.07	-	-	-	-	-
LMM	-		-	-	-	-	-	-	-	4.53
LOL	-	-	-	-	11.30–12.55	-	-	-	-	-
LOM	-	-	-	-	5.80–6.54	-	-	-	-	-
LOP	-	-	-	-	3.32–3.54	-	-	-	-	-
LOS	-	-	-	-	1.66–1.68	-	-	-	-	-
LLnL	-	-	-	-	2.10–2.13	-	-	-	-	-
LLnM	-	-	-	-	1.07–1.11	-	-	-	-	-
LLnP	-	-	-	-	0.60–0.62	-	-	-	-	-
LLiL	-	-	-	-	0.40	-	-	-	-	-
LaLaP	-	-	-	0.4	-	-	-	-	-	-
LaMP	-	-	-	0.9	-	-	-	-	-	-
LaOL	-	-	-	1.8	-	-	-	-	-	-
LaOLa	-	-	-	0.8	-	-	-	-	-	-
LaPP	-	-	-	1.2	-	-	-	-	-	-
LaOM	-	-	-	3.2	-	-	-	-	-	-
LaLM	-	-	-	1.5	-	-	-	-	-	-
MML	7.85	-	14.62	-	-	-	-	-	-	-
MOO	-	-	-	3.9	-	-	1.2	-	-	-
MOL	-	-	-	3.5	-	-	-	-	-	-
MOS	-	-	-	-	0.41–0.42	-	-	-	-	-
MOP	-	-	-	10.4	-	-	-	-	-	-
MPP	-	-	-	1.9	-	-	-	-	-	-
MOM+LaOP	-	-	-	7.9	-	-	-	-	-	-
MLM	-	-	-	3.3	-	-	-	-	-	-
MLP+LaOO	-	-	-	6.7	-	-	-	-	-	-
PPL	-	-	-	-	1.43–1.65	-	-	-	-	-
PPLn	-	-	-	-	-	-	1.0	-	-	-
PPO	-	-	-		-	-	7.4			-
PPoP	-	-	-	-	-	3.7	-	-	-	-
PPP	-	1.8	-	1.6	-	3.1	-	-	0.9	-
PLM	7.28	-	7.50	-	-	-	-	-	-	-
PLS	-	1.2	-	0.9	-	1.6	-		4.0	-
PLL	-	3.3	-	1.6	-	-	0.6	-	5.9	-
PLP	-	1.1	-	5.7	-	-	-	-	8.3	-
POL	-	-	-	6.2	-	6.6	2.5–3.9	-	17.7	-
POLn	-	-	-	-	-	-	3.9	-	-	-
POP	-	22.5	-	11.5	-	32.4	9.4	-	10.3	-
POPo	-	-	-	-	-	12.2	-	-	-	-
POO	-	38.8	-	12.5	-	28.5	25.1–38.8	-	19.0	-
POS	-	3.4	-	-	-	2.1	1.3	-	3.1	-
OLL	-	-	-	-	-	-	-	-	2.8	-
OLLn	-	-	-	-	-	-	1.6	-	-	-
OLnL	-	-	-	-	0.80	-	-	-	-	-
OLnLn	-	-	-	-	-	-	1.4	-	-	-
OOP	-	-	-	-	-	-	-	19.27	-	-
OOL	-	-	-	2.1	-	0.8	1.7–6.9	-	8.0	-
OOLn	-	-	-	-	-	-	11.1	-	-	-
OOLi	-	-	-	-	-	-	-	9.53	-	-
OOO	-	19.4	-	3.9	-	6.1	28.8–35.6	38.83	9.3	-
OOS	-	-	-	-	-	-	-	7.95	-	-
SLL	-	2.4	-	-	-	-	-	-	2.1	-
SLP	-	-	-	-	0.53–0.64	-	-	-	-	-
SOO	-	3.3	-	0.8	-	1.1	1.6–3.5	-	2.4	-
SOL	-	1.1	-	-	-	-	2.3	-	4.0	-
References	[10]	[148]	[10]	[148]	[138]	[148]	[148,172]	[10]	[148]	[69]

* Mass fraction (wt%). C, capric acid; Cp, caprylic acid; L, Lauric acid; M, myristic acid; O, oleic acid; P, palmitic acid; Po, palmitoleic acid; Li, linoleic acid; Ln, linolenic acid; S, stearic acid. Acronyms: MUK, murumuru kernel butter (*A. murumuru*); TPP and TPK, tucumã-do-Pará pulp and kernel oils (*A. vulgare*); INP, inajá pulp oil (*A. maripa*); BBK, babassu kernel oil (*A. speciosa*); PUP, peach palm pulp oil (*B. gasipaes*); BRP, buriti pulp oil (*M. flexuosa*); PTP, patawa pulp oil (*O. bataua*); BAP, bacaba pulp oil (*O. bacaba*); LIK, licuri kernel oil (*S. coronata*).

## Data Availability

Not applicable.
